# Fungal-specific PCR primers developed for analysis of the ITS region of environmental DNA extracts

**DOI:** 10.1186/1471-2180-5-28

**Published:** 2005-05-18

**Authors:** Kendall J Martin, Paul T Rygiewicz

**Affiliations:** 1 Dynamac Corporation, National Health and Environmental Effects Research Laboratory, Corvallis, OR USA; 2 USEPA National Health and Environmental Effects Research Laboratory, Corvallis, OR, USA

**Keywords:** PCR, primers, basidiomycetes, ascomycetes, Internal Transcribed Spacer (ITS), X/T rapid extraction method

## Abstract

**Background:**

The Internal Transcribed Spacer (ITS) regions of fungal ribosomal DNA (rDNA) are highly variable sequences of great importance in distinguishing fungal species by PCR analysis. Previously published PCR primers available for amplifying these sequences from environmental samples provide varying degrees of success at discriminating against plant DNA while maintaining a broad range of compatibility. Typically, it has been necessary to use multiple primer sets to accommodate the range of fungi under study, potentially creating artificial distinctions for fungal sequences that amplify with more than one primer set.

**Results:**

Numerous sequences for PCR primers were tested to develop PCR assays with a wide range of fungal compatibility and high discrimination from plant DNA. A nested set of 4 primers was developed that reflected these criteria and performed well amplifying ITS regions of fungal rDNA. Primers in the 5.8S sequence were also developed that would permit separate amplifications of ITS1 and ITS2. A range of basidiomycete fruiting bodies and ascomycete cultures were analyzed with the nested set of primers and Restriction Fragment Length Polymorphism (RFLP) fingerprinting to demonstrate the specificity of the assay. Single ectomycorrhizal root tips were similarly analyzed. These primers have also been successfully applied to Quantitative PCR (QPCR), Length Heterogeneity PCR (LH-PCR) and Terminal Restriction Fragment Length Polymorphism (T-RFLP) analyses of fungi. A set of wide-range plant-specific primers were developed at positions corresponding to one pair of the fungal primers. These were used to verify that the host plant DNA was not being amplified with the fungal primers.

**Conclusion:**

These plant primers have been successfully applied to PCR-RFLP analyses of forest plant tissues from above- and below-ground samples and work well at distinguishing a selection of plants to the species level. The complete set of primers was developed with an emphasis on discrimination between plant and fungal sequences and should be particularly useful for studies of fungi where samples also contain high levels of background plant DNA, such as verifying ectomycorrhizal morphotypes or characterizing phylosphere communities.

## Background

Studies of fungi (Kingdom Eumycota) in natural environments often require simultaneous analysis of a broad taxonomic range. For instance, the fungi forming ectomycorrhizal symbioses number over 5000 species [1]. Analysis of natural ectomycorrhizal fungal communities traditionally has been a laborious, highly-skilled process with heavy reliance on gross morphological characterization of the ectomycorrhizal root-tips. The laborious nature of microscopic analysis and identification is the driving force behind developing such methods as molecular verification of identified morphotypes by means of PCR-RFLP [2]. Analysis of natural populations of ectomycorrhizas requires high rates of sampling because of the high number of fungal species involved and the high spatial variability observed in natural systems [3,4]. Primers allowing simultaneous analysis of all the fungal phyla which are involved in ectomycorrhizal symbioses would be a useful tool in studying the ecology of these fungi. Of particular interest for such work is the division Dikaryomycota, which includes the subdivisions Basidiomycotina and Ascomycotina, and encompasses all ectomycorrhizal fungi [1].

The earliest PCR primers to gain wide acceptance for work with fungal Internal Transcribe Sequences (ITS) were "ITS1" and "ITS4" which amplify the highly variable ITS1 and ITS2 sequences surrounding the 5.8S-coding sequence and situated between the Small SubUnit-coding sequence (SSU) and the Large SubUnit-coding sequence (LSU) of the ribosomal operon [5]. These primers amplify a wide range of fungal targets and work well to analyze DNA isolated from individual organisms, but do not exclude effectively the plant host sequences in mixed, phytosphere DNA extracts typical of studies of plant-associated microbiota. Subsequently, the plant-excluding primers ITS1-F and ITS4-B came into wide use for analyses of fungal ITS sequences, but these primers were "intended to be specific to fungi and basidiomycetes, respectively" [6]. We present here a suite of primers designed to amplify Dikaryomycota efficiently, requiring little optimization for use in the hands of even relatively untrained operators.

In addition to the increased efficiency needed for well-established methods such as PCR-RFLP analyses of single fungal species [7], robust primers are needed for newer molecular methods currently used to characterize microbial communities: Length Heterogeneity PCR (LH-PCR) [8], high-throughput sequencing [9], and Terminal Restriction Fragment Length Polymorphism (T-RFLP) [10]. LH-PCR is a reliable and effective approach to analyze targets with high variability in overall length. In T-RFLP analyses, as with LH-PCR, a fluorescent label on the PCR primer is used for detection, but in this case only the primer-terminal fragments of restriction digested PCR products are detected. These fragments contain the labelled primer and extend to the first instance of a restriction site for the enzyme used. With increased access to capillary-electrophoresis instruments capable of high resolution discrimination of oligonucleotide lengths, these methods have become both rapid and reliable. Such methods enable rapid analysis of environmental samples and can provide extensive data on microbial communities as defined or restricted by the specificity range of primers used. These data include both relative abundances of dominant microbial phylotypes and characteristic PCR-product or TRF (terminal restriction fragment) sizes for these phylotypes. For comparison of fungal communities, either method provides a relatively complete, culture-independent analysis. Additionally, where the effort is justified, identified phylotypes subsequently can be taxonomically characterized (by applying sequence analysis to amplified targets) with the advantage that resources can be focused on phylotypes that are most important to the study at hand. Terminal restriction fragment sizes also can be compared to a database of theoretical restriction fragments derived from sequence information to approximate taxonomic identity [11,12].

While LH-PCR or T-RFLP gives an estimate of relative abundance of phylotypes in a community, quantitative PCR (QPCR) gives an overall quantification for the target sequence which can then be subdivided mathematically to provide an estimate of absolute population level for individual phylotypes. Higuchi *et al. *[13,14] developed a method for real-time detection of PCR products to quantify sample target sequences based on fluorescence of the intercalating dye ethidium bromide, and numerous companies now offer kits for such quantification using SYBR Green I (Invitrogen Life Technologies, Carlsbad, CA) as the intercalating dye. Most of these instruments now include the ability to perform a melting curve analysis on the PCR product after quantification [15], which partially ameliorates uncertainty arising from the inability of the dye to distinguish target amplicons from non-target PCR products.

We describe a set of primers which performs well in all of these analytical approaches. This characteristic of wide applicability to studies of Dikaryomycota from diverse environmental samples, particularly those with significant plant tissue content, lends a degree of interoperability between analytical approaches. For instance, real-time PCR analysis of samples can guide the choice of cycle numbers for LH-PCR or T-RFLP reactions which are sensitive to late stage PCR. This is important for comparing data between more established approaches and newer methods coming into popular use.

## Results and discussion

### Initial work with fungal PCR

**Figure 1:**
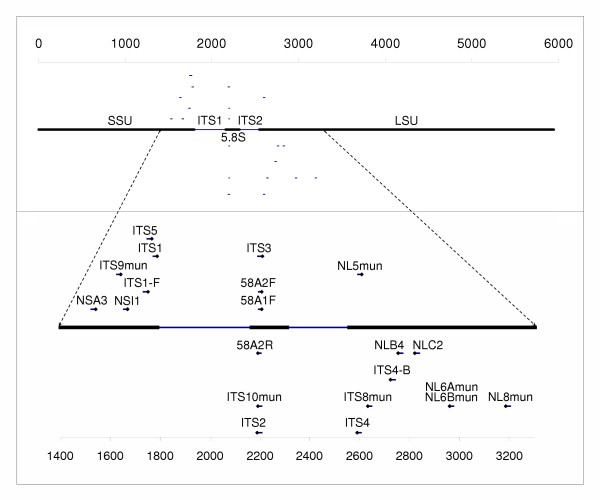
**Diagram of primer locations in the ribosomal cassette consisting of SSU, ITS1, 5.8S, ITS2, and LSU rDNA**. Primers are positioned above (forward primers) or below (reverse) their sequence positions. ITS1, ITS2, ITS3, and ITS4 from White *et al*. [5], primers ITS8mun, ITS9mun, ITS10mun, NL5mun, NL6Amun, NL6Bmun, NL8mun from Egger [16], primers ITS1-F, ITS4-B from Gardes and Bruns [6] and the remaining primers (NSA3, NSI1, 58A1F, 58A2F, 58A2R, NLB4, NLC2) from this study. Scale is in base pairs according to the extension of the Gargas and DePriest [23] nomenclature system described in this study.

We began investigating fungal ITS primers using ITS1-F as the forward primer situated at the 3' end of SSU and ITS4-B as the reverse primer in the 5' section of LSU [6]. These primers amplify the entire ITS region (Figure 1). The reverse primer, ITS4-B, was not intended to amplify ascomycete targets, however, and based on sequence comparisons, it appears that it can be a poor match to many basidiomycetes. We also investigated reverse primers in the 5' section of LSU developed by Egger [16]. These primers separately amplify Ascomycetes and Basidiomycetes. All of these primers have strong positive attributes, but since our morphological analysis of ectomycorrhizal root tips did not distinguish between Ascomycetes and Basidiomycetes, we developed primers that were capable of amplifying all Dikaryomycota simultaneously.

Initially, we approached our goal of a reliable PCR amplification broadly targeting Dikaryomycota by developing a primer (NLB3) which works well with ITS1-F and is close to the annealing site for ITS4-B. The ITS1-F/NLB3 primer pair effectively excludes plant sequences, amplifies basidiomycete targets, and additionally, amplifies ascomycete targets. We found, however, that ITS1-F apparently caused spurious product bands from low concentration (single root tip) DNA extracts of our ectomycorrhizas. The source of these bands was verified by reamplification with ITS1-F alone. The ITS1-F/NLB3 primer pair has been shown to be suitable for mycorrhizal applications in another laboratory [17].

### Fungal ITS primer development

In order to obtain a cleaner product for restriction, we developed an alternative to ITS1-F, resulting in a primer pair which shows greater specificity to the intended fungal ribosomal target (Figure 1). We also developed a robust pair of Dikaryomycota-specific primers (NSA3/NLC2) that could serve as first round primers in nested-PCR reactions with annealing sites outside those for a second pair of Dikaryomycota-specific primers (NSI1/NLB4). We have used this nested reaction extensively for PCR-RFLP work with ectomycorrhizal root tips. The NLB4 primer is identical to the NLB3 primer except that it has one additional base at the 3' end (see Table 1) and has a slightly higher calculated melting temperature.

**Figure 2:**
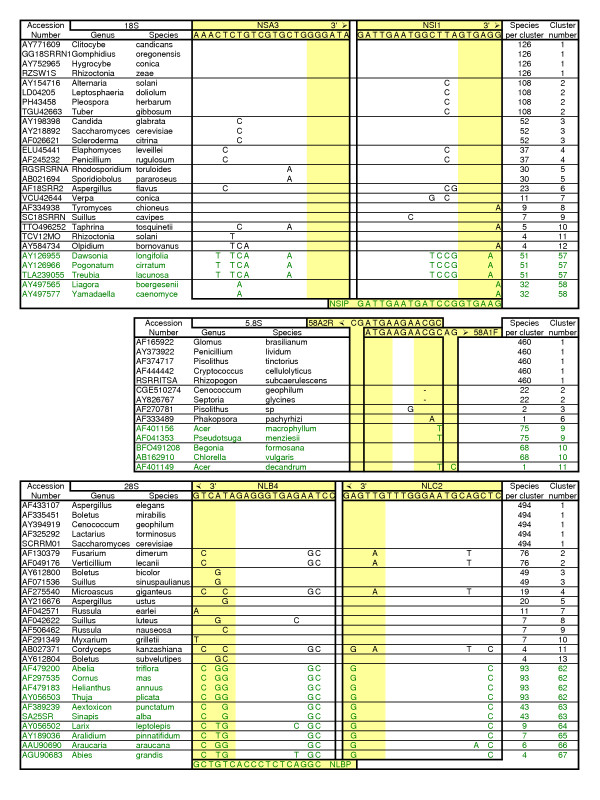
**Multiple alignments of published sequences for the primer sites**. Primer sequence alignments are depicted with consensus bases blank and mismatch bases noted. The six bases at the 3' end of the primers are highlighted yellow. "Clusters" were created by sorting the sequences in Excel and grouping identical sequences. "Species per cluster" numbers indicate the number of species with that mismatch pattern. Only one sequence per species was included in the alignment (final totals of: 584 species for 18S, 633 species for 5.8S, and 943 species for 28S). The "Cluster number" is a sequential numbering of clusters in order of descending "Species per cluster" with sequences from the EMBL Fungal database numbered before sequences from the EMBL Plant database (the latter are presented in green font). Sequences were collected using FASTA [40] for a set of 12 taxonomically representative fungal sequences for each region. Most mismatch groups containing fewer than 4 species were removed from the figure to save space. The full alignment is also available [see Additional file 1].

Additional primer development allowing for separate amplification of ITS1 and ITS2 produced a final suite of 6 primers (see Table 1, Figure 1) specific to Dikaryomycota: outer nested-PCR primers (NSA3/NLC2), a pair of primers that amplify both ITS regions (NSI1/NLB4) and forward (58A1F, 58A2F) and reverse (58A2R) primers in the 5.8S sequence (overlapping with ITS3/ITS2 of White *et al. *[5]) for amplification of internal transcribed spacers ITS2 and ITS1, respectively. The outer nested-PCR primers (NSA3/NLC2) work well with all other fungal primer pairs (Figure 1) in this suite and provide greater sensitivity and specificity (and can be used to verify the identity of the product). Two primers specific to Plantae were also developed (NSIP/NLBP), and for our research can be used to verify that fungal PCR product is not contaminated by plant sequences (Table 1, Figure 2 and see Additional File 1). These primers are situated at the best estimate of the positions homologous to the fungal primers, NSI1 and NLB4, by multiple sequence alignment. The plant primers have been applied successfully to LH-PCR and PCR-RFLP analyses of forest plant tissues from above- and below-ground samples and work well at discriminating a representative sampling of plants from a forest in the Cascade Mountains in Oregon USA to the species level. Length heterogeneity was sufficient to distinguish more than 20 understory species (data not shown) in an old-growth Douglas-fir forest *(Pseudotsuga menziesii *(Mirbel) Franco), but the conifers [Douglas-fir, silver fir (*Abies amabilis *(Dougl.) Forbes), and hemlock (*Tsuga heterophylla *(Raf.) Sarg.)] required PCR-RFLP to obtain species level identification.

To ensure that the suite of fungal primers was amplifying the intended target sequences, fungal PCR products were verified in three ways. Firstly, PCR-RFLP patterns obtained under the current PCR protocol were compared to known patterns derived from sequences for the *Saccharomyces cerevisiae *ribosomal operon (SCYLR154C, Z73326, *S. cerevisiae *chromosome XII) using three restriction enzymes to ensure that fidelity was maintained. Secondly, PCR-RFLP patterns for ITS1 or ITS2 PCR reactions were checked against the same patterns derived from a nested PCR starting with the NSA3/NLC2 first-round primer pair. In this case, insufficient genomic DNA was carried over to the second round to amplify on its own so the product of the inner, second-round primers must have arisen from target sequences within the first product and not from another part of the genome. Thirdly, for DNA extracts with high percentages of plant DNA, the plant primers NSIP and NLBP were used to characterize the product and/or patterns that would result from amplifying plant sequences because of insufficient stringency. These methods were also incorporated into a quality assurance protocol.

### Efficacy of the new fungal PCR system

**Figure 3:**
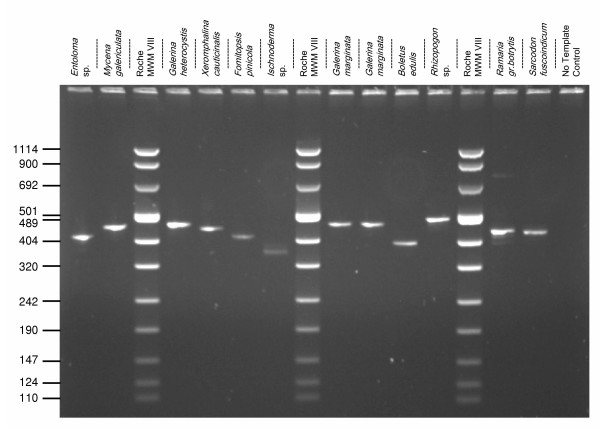
**Example of the nested NSI1/NLB4 PCR reaction applied to single-species fungal DNA extracts**. Ethidium Bromide fluorescence image showing electrophoresis of nested NSI1/NLB4 PCR product for a group of sporocarp extracts. Gel was run with 0.7% high melting point agarose plus 2% Synergel additive (Diversified Biotech, Boston, MA, cat. no. SYN-100). Roche DNA molecular weight marker VIII (cat. no. 1336045) was loaded at 100 ng in lanes 3, 8 and 13 (sizes for fragments to the left of the figure). Ten μL of PCR reaction was loaded in each well for each of the templates listed at the top of the gel as well as the no-template control in the last well.

**Figure 4:**
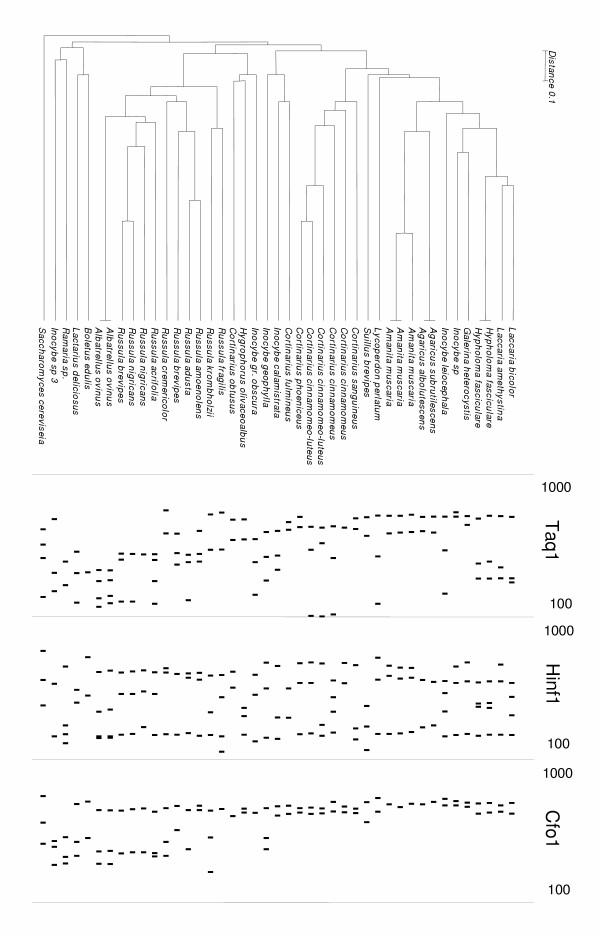
**Dendrogram and PCR-RFLP patterns for sporocarps**. The upper section shows the species names and the cluster tree resulting from presences/absence analysis of the PCR-RFLP data. PCR-RFLP fragment sizes are presented below in base pairs for *Taq1*, *HinF1 *and *Cfo1 *restrictions as indicated at the right of the figure.

The primer pair NSI1/NLB4 has successfully amplified 32 species of fungal sporocarps in 15 genera collected from geographically diverse locales in Oregon (all basidiomycetes but *Tuber*). As can be seen in Figure 3, the high diversity of the ITS sequences is evident from the variation in PCR product lengths. These amplifications were used to perform PCR-RFLP cluster analysis (Figure 4). The primer pair NSI1/NLB4 also has successfully amplified 26 species in 15 genera of dilution-plate isolated ascomycetous soil microfungi. No *Zygomycetes glomus *species were tested and from sequence data it appears unlikely that they would amplify (Figure 2). The mycorrhizal Ascomycetes, *Cenoccocum *(identified visually and by the presence of the ITS1 intron [18]) and *Tuber melanosporum*, have amplified well and given restriction patterns consistent with their published sequences (Table 2). The ITS1 intron is also found in *Hymenoscyphus ericae *[19]. Occurrence of this ITS1 intron may be widespread enough to affect some studies where the large size of the amplicons (800–1000 base pairs) causes technical difficulties. If necessary, the forward primer, ITS1 [5], may be used with NLB4 as an alternative since this non-specific primer is downstream of the intron and can be used to avoid amplifying the intron.

The capacity of these primers to amplify fungal targets has encouraged us to develop numerous collaborations where the primers have been applied to a wide range of techniques. Examples of applying the primers in current projects include:

1) amplified ectomycorrhizal fungi from more than 2000 single-root-tip DNA extracts [20],

2) the primer pair NSI1/NLB4 has successfully amplified 30 species in 18 genera of plate-isolated ascomycetous soil microfungi (identified by Drs. Lidia Watrud and Jeffrey Stone, personal communication),

3) PCR-RFLP, T-RFLP and direct-sequencing analyses of individual morphotypes and whole-core extracts of ectomycorrhizal fungi on Loblolly pine grown in North Carolina USA [21] (Burke *et al*, personal communication),

4) characterized ectomycorrhizal fungi found on roots of eucalypts grown in Uruguay [17],

5) analysis of epiphytic fungi on agricultural crops by QPCR and LH-PCR [22].

Of the two primers situated in 5.8S, 58A1F gives slightly more robust PCR reactions than 58A2F, but based on sequence comparisons, is more likely to have difficulty amplifying *Cenococcum *targets. Both work well with NLB4, the LSU reverse primer used for ITS2 analysis. They both lay within the sequence for ITS3 [5] and are shorter by two bases: 58A1F being shorter on the 5' end and 58A2F being shorter on the 3' end.

### Xanthogenate/Tween rapid extraction method

In this study we also evaluated possible increases in efficiency of analyses by using the Xanthogenate/Tween (X/T) rapid extraction method instead of the CTAB/chloroform extraction. We initially tested the X/T method with additions of DNase to confirm the ability of the solution to protect DNA (commercially available *S. cerevisiae *genomic DNA, Promega cat. no. G3101). Comparing agarose gels showed that DNA degradation or loss attributable to DNase were not detectable; the same result was obtained with the CTAB extraction. DNA yields from single root tips using X/T were sufficient to give useful PCR reactions for more than half of the samples analyzed. Around 80% of single root tips extracted by the CTAB method yielded amplifiable fungal DNA using the nested amplification presented in this study. There are approximately half as many transfers in the X/T method as in the CTAB method and fewer centrifugations, so we are able to process four times as many samples in the same time. The fungal tissue of the ectomycorrhizas we were studying is concentrated mostly on the outside of the ectomycorrhizal root tips. By not grinding the samples, and therefore extracting relatively more from the surface than the interior of the ectomycorrhizal root tip, we also decrease the amount of plant DNA and phenolics in the extracts. Consequently, for the analyses where the goal was PCR-RFLP verification of ectomycorrhizal roots first classified using gross morphological traits, only the more healthy mycorrhizas would likely produce strong patterns for the primary fungal symbionts. This was seen potentially as a positive attribute of the X/T extraction procedure. Those root tips that failed to yield amplifiable fungal DNA by the X/T procedure may have done so after CTAB extraction, but also may be senescent roots colonized by saprobic fungi. In other words, the high sensitivity of the CTAB extraction in conjunction with the nested PCR carries an increased risk of identifying saprobic fungi as ectomycorrhizal fungi where the ectomycorrhizal fungi are absent, senescent or otherwise poorly amplifiable. It may be that the decreased sensitivity of the X/T extraction procedure along with potential preferential extraction of DNA from healthy mycorrhizal fungi would help to overcome this risk. Researchers should bear in mind, though, that this bias towards root-surface fungal tissue could lead to underestimating fungal types that primarily reside within the root. The Xanthogenate/Tween extraction procedure should be seen as a complement to extractions involving tissue homogenization. The combination of these approaches could allow for some interesting possibilities such as localizing fungal distribution on and in the root.

## Conclusion

The new suite of primers for Dikaryomycota presented here (specified using an extension of the Gargas and DePriest [23] numbering system) is anticipated to serve as the basis for a wide ranging system to analyze microbial communities, particularly in association with plants. In addition to the new plant primers, we are developing homologous primer sets for Oomycota and Zygomycota. By using the same sequence positions for multiple primer sets, we expect to maintain a high degree of comparability between PCR reactions. This approach also allows us to use an iterative approach to overcoming difficulties inherent in defining the taxonomic range of any particular primer set by developing "homologous" primers with differing target ranges. Emergent methods for molecular analysis of microbial communities are allowing for increasingly high sampling rates. Increased sampling rates are, in turn, the driving force behind more effectively characterizing these spatially-diverse and complex groups. Methods such as the Xanthogenate /Tween DNA extraction procedure similarly allow for rapid screening of large numbers of samples. Future studies of microbial communities are likely to rely heavily on methods that can be adapted to large-scale sample processing needed to make efficient use of increasingly rapid and powerful molecular technologies.

## Methods

### Primer description

Primers were developed using multiple sequence alignments [24] of a representative taxonomic range of fungal and plant sequences downloaded from EMBL [25]. Nomenclature developed by Gargas and DePriest [23] for fungal primers was used with the exception that the sequence they refer to covers only the 1798 bases of 18S. We used the complementary sequence for *Saccharomyces cerevisiae *chromosome XII (EMBL ID SCYLR154C) to extend the numbering beyond the final base of 18S (EMBL ID SCRGEA). In this extension, the complement to base 8356 of SCYLR154C is the final base, 1798, of SCRGEA. The resulting *S. cerevisiae *ribosomal operon numbering system can be calculated by subtracting the SCYLR154C base-position from 8356 and adding this difference to the 1798 bases of SCRGEA.

### Test samples

Soil microfungi from litter and soil in the Cascade Mountains in Oregon USA were isolated on DRB medium [26] and were subcultured to malt agar and Czapek's with yeast extract [27] for microscopic examination. Isolates were identified on the basis of conidium formation and conidiophore structure [27-30]. Sporocarps were collected and archived from numerous locations in Oregon. They were identified by gross morphology and spore color [31,32]. Ectomycorrhizal root tips were sorted based on gross morphological features (*e.g.*, branching pattern, color, mantle texture, size, shape of tip, luster) as described elsewhere [33].

### DNA extraction

For most routine extractions of fungal tissue and our early extractions of ectomycorrhizal root tips, DNA was extracted using a CTAB protocol with 0.8% mercaptoethanol and incubating at 65°C for 1 hour [34]. An alternate extraction method modified from Ross [35] based on the action of xanthogenate [36] was later employed for rapid survey analyses of large numbers of ectomycorrhizal root tips. Single ectomycorrhizal root tips (approximately 1 mg tissue dry weight) were placed in each 1.5 mL tube with 600 μL Xanthine/Tween Buffer (100 mM Tris-HCl pH 7.5, 12.5 mM potassium ethyl xanthogenate (Fluka, Buchs, Switzerland, cat. no. 60040), 10 mM EDTA pH 8.0, 10% Tween 20, 500 mM NaCl). These were sonicated for 15 seconds and incubated 90–120 min at 60°C on a shaker. Tubes were then centrifuged 5 min at 10,000 × g to pellet undissolved tissue. Supernatant was removed to a new tube containing 1/5 volume of PEG/NaCl (20% PEG-8000/2.5 M NaCl [37]) and 8 μL 1.25 mg/mL Linear Acrylamide (Ambion, Austin, Texas, cat. no. 9520). This was mixed by gently tipping the tube, and the mixture was incubated at 30°C for 15 minutes. DNA was precipitated by centrifuging 5 minutes (room temp.) at 10,000 × g, and then recovered by removing the PEG solution. The final DNA pellet was obtained by rinsing twice with 200 μL 80% ice-cold ethanol while mixing by gently tipping the tubes, followed by centrifuging 5 minutes at 10,000 × g, and lastly, by drying the pellet under vacuum for 30–45 minutes. In both protocols DNA was resuspended in a final volume of 100 μL TE buffer (pH 8, 10 mM Tris: 1 mM EDTA) after extraction by heating to 37°C for 10 minutes.

### PCR amplification

PCR was carried out in 50 μL reaction volumes using 2.0 mM MgCl_2_, 0.2 μM each primer, 0.2 mM dNTP, 0.5 mg [mL]^-1 ^bovine serum albumin (BSA) and 0.04 U [μL]^-1 ^FastStart Taq DNA polymerase (Roche Diagnostics GmbH, Mannheim, Germany) on a thermal cycler equipped with a heated lid. An initial denaturation and enzyme activation step of 6–10 minutes at 95°C was followed by amplification for 35 cycles at the following conditions: 30 seconds at 95°C, 40 seconds at 60°C, 40 seconds at 72°C. A final 5-minute extension at 72°C completed the protocol. PCR typically yielded between 50–60 ng amplicon [μL]^-1^.

### Restriction analysis

PCR-RFLP for the ITS region was performed with three restriction endonucleases: *Cfo*I, *Hinf*I, and *Taq*I. After electrophoretic separation on 3% agarose gels containing ethidium bromide, images were captured using the AlphaImager 950, version 3.24 (Alpha Innotech Corporation, San Leandro, CA), image capture system and analyzed using the Gene Profiler program (Scanalytics, Inc., Fairfax, VA). After restriction patterns were quantified using Gene Profiler, data were collected in the associated database facility to generate a presences/absence binary-data file. This file was input to TreeConW software [38] to develop dendrograms using the Link algorithm [39], {Gdxy = (Nx + Ny) / (Nx + Ny + Nxy)}, and "Unweighted Pair-Group Method using Arithmetic averages" (UPGMA) clustering.

### DNA quantification

Absolute amounts of target DNA sequences extracted from samples were quantified by real-time PCR (QPCR) employing the DNA-binding fluorophore SYBR^® ^Green 1 (Invitrogen Life Technologies, Carlsbad, CA). QPCR was performed on a RotorGene 3000 centrifugal amplification system (Corbett Research, Mortlake, Australia). PCR was performed with the primer pair, NSI1/58A2R, in 15 μL reaction volumes containing between 10 ng and 10 pg purified DNA, 0.40x SYBR^® ^green, 0.50 mg mL^-1 ^BSA, 3.0 mM MgCl, 0.6 μm each primer, 0.2 mM dNTP, and 0.1 U [μL]^-1 ^FastStart Taq DNA polymerase in 100 μL tubes. A dilution series containing known amounts of *S. cerevisiae *genomic DNA (Promega Corporation, Madison, WI, cat. no. G3101) was used as the standard for quantification of sample DNA in each morphotype class. Upon completing PCR, melting curve analysis was used to determine whether there was detectable primer-dimer contribution to the SYBR green fluorescence measurement of amplified DNA (identified as a distinct drop in fluorescence with increasing temperature at temperatures below 82°C where low-melting-point, non-target amplicons denature). Samples compromised by primer-dimer reactions were deleted from the analysis. Using a value of 17 femtograms per haploid genome of *S. cerevisiae*, we calculated *Saccharomyces *genome equivalents (SGE) per μL of extract volume.

## Abbreviations

BSA (Bovine Serum Albumin), ITS (Internal Transcribed Spacer), LH-PCR (Length Heterogeneity PCR), LSU (Large SubUnit-coding sequence), PCR-RFLP (Polymerase Chain Reaction-Restriction Fragment Length Polymorphism), QPCR (Quantitative PCR), SSU (Small SubUnit-coding sequence) TRF (Terminal Restriction Fragment), T-RFLP (Terminal Restriction Fragment Length Polymorphism), X/T (Xanthogenate/Tween rapid extraction method)

## Authors' contributions

Dr. Kendall J. Martin primarily was responsible for the research direction, conducting the research, and interpreting and presenting the results. Dr. Paul T. Rygiewicz instituted and coordinated the overarching research projects within which this work was hosted and played an advisory role in pursuing this research and developing the manuscript.

## Supplementary Material

Additional File 1 **Multiple alignments of published sequences for the primer sites**. This is the full set of sequences aligned to show the range of potential compatibility for the primers. This set was greatly reduced to create Figure 2. Click here for file

## Figures and Tables

**Table 1:** Sequence characteristics of primers developed in this study.

**Name**	**Sequence (5' to 3')**	**Nomenclature**	**Bases**	**Tm**
NSA3	AAACTCTGTCGTGCTGGGGATA	nu-SSU-1543-5'	22	67
NSI1	GATTGAATGGCTTAGTGAGG	nu-SSU-1671-5'	20	59
58A1F	GCATCGATGAAGAACGC	nu-5.8S-2206-3'	17	61
58A2F	ATCGATGAAGAACGCAG	nu-5.8S-2208-3'	17	57
58A2R	CTGCGTTCTTCATCGAT	nu-5.8S-2192-5'	17	57
NLB3	GGATTCTCACCCTCTATGA	nu-LSU-2754-5'	19	56
NLB4	GGATTCTCACCCTCTATGAC	nu-LSU-2755-5'	20	57
NLC2	GAGCTGCATTCCCAAACAACTC	nu-LSU-2821-5'	22	67
Plant control primers				
NSIP	GATTGAATGATCCGGTGAAG	PC-nu-SSU-1671-5'	20	62
NLBP	GCTGTCACCCTCTCAGGC	PC-nu-LSU-2755-5'	18	64

Sequences are all listed 5' to 3' for the oligonucleotides. Nomenclature after Gargas and DePriest [23] as extended in this study. Tm is calculated by nearest neighbour method as an on-line service provided by Sigma-Genosys (The Woodlands, TX).
