# Supplementary material for: Fungal-specific PCR primers developed for analysis of the ITS region of environmental DNA extracts
Source: BMC Microbiol. 2005 May 18;5:28. doi: 10.1186/1471-2180-5-28 (PMC1156903; doi:10.1186/1471-2180-5-28)
Supplement: Additional File 1 — Multiple alignments of published sequences for the primer sites. This is the full set of sequences aligned to show the range of potential compatibility for the primers. This set was greatly reduced to create Figure 2. [file 1471-2180-5-28-S1.pdf]

Accession	18S		NSA3																					3' end		NSI1																			3' end	Speceis	Cluster
Number	Genus	Species	A	A	A	C	T	C	T	G	T	C	G	T	G	C	T	G	G	G	G	A	T	A	-	G	A	T	T	G	A	A	T	G	G	C	T	T	A	G	T	G	A	G	G	per cluster	number
AY771609	Clitocybe	candicans																																												126	1
GG18SRRN1	Gomphidius	oregonensis																																												126	1
AY752965	Hygrocybe	conica																																												126	1
RZSW1S	Rhizoctonia	zeae																																												126	1
AY154716	Alternaria	solani																																		C								108	2
LD04205	Leptosphaeria	doliolum																																		C								108	2
PH43458	Pleospora	herbarum																																		C								108	2
TGU42663	Tuber	gibbosum																																		C								108	2
AY198398	Candida	glabrata							C																																			52	3
AY218892	Saccharomyces	cerevisiae							C																																			52	3
AF026621	Scleroderma	citrina							C																																			52	3
ELU45441	Elaphomyces	leveillei					C																													C								37	4
AF245232	Penicillium	rugulosum					C																													C								37	4
RGSRSRNA	Rhodosporidium	toruloides														A																												30	5
AB021694	Sporidiobolus	pararoseus														A																												30	5
AF18SRR2	Aspergillus	flavus					C																													C	G							23	6
VCU42644	Verpa	conica																																G		C								11	7
AF334938	Tyromyces	chioneus																																									A	9	8
SC18SRRN	Suillus	cavipes																													C													7	9
TTO496252	Taphrina	tosquinetii							C							A																											A	5	10
TCV12MO	Rhizoctonia	solani						T																																				4	11
AY584734	Olpidium	bornovanus						T	C	A																																	A	4	12
GLA301970	Glomerella	lagenaria						G																												C								3	13
AF518594	Vuilleminia	comedens					C																																					3	14
AF184757	Pilophorus	robustus							G																											C								3	15
AY188389	Bullera	siamensis							A																									T									A	3	16
AB178481	Sporobolomyces	gracilis							G							A																												3	17
AY635833	Glomus	mosseae						T	C	A						A																											A	3	18
AY154706	Alternaria	arborescens																																		C								2	19
AY154705	Alternaria	citri																																		C								2	20
AB085797	Cryptococcus	flavescens							A																												G						A	2	21
BAD323	Bullera	armeniaca							A																																		A	2	22
BVSSRRGG	Bullera	variabilis							A							A																		T									A	2	23
AY771603	Dacryopinax	spathularia						T	C																																			2	24
AY635839	Oedogoniomyces	sp						T	C	A																																		2	25
AY635841	Basidiobolus	ranarum				T		T	C	A														T																			A	2	26
AB047309	Glomus	leptotichum						T	C	A																					C												A	2	27
AY126955	Dawsonia	longifolia				T		T	C	A						A																		T	C	C	G					A		51	57
AY126966	Pogonatum	cirratum				T		T	C	A						A																		T	C	C	G					A		51	57
TLA239055	Treubia	lacunosa				T		T	C	A						A																		T	C	C	G					A		51	57
AY497565	Liagora	boergesenii							A																																		A	32	58
AY497577	Yamadaella	caenomyce							A																																		A	32	58
RH18SRRN	Reboulia	hemisphaerica				A		T	C	A						A																		T	C	C	G					A		3	59
AF141970	Opalina	ranarum				T		T	C	A																								T									C	2	60
RSP7286	Rhodomonas	sp				A		T	C	A						A																		T	C	C	G					A	A	2	61
TAM7287	Teleaulax	amphioxeia				G		T	C	A						A																		T	C	C	G					A	A	2	62
DSU16520	Dawsonia	superba				T		T	C	A						A						G												T	C	C	G					A		2	63

Accession Number	18S Genus	Species	NSA3 A A A C T C T G T C G T G C T G G G G A T A	-	NSI1 G A T T G A A T G G C T T A G T G A G G	Speceis per cluster	Cluster number
AF334899	Abortiporus	biennis				126	1
AY445116	Agaricales	sp				126	1
ABRGDA	Agaricus	bisporus				126	1
AY707091	Albatrellus	higanensis				126	1
U53371	Aleuria	aurantia				126	1
AY550243	Amanita	bisporigera				126	1
AF026631	Amanita	muscaria				126	1
AF518570	Amylocystis	lapponica				126	1
AY378095	Antrodia	camphorata				126	1
ALY496254	Asterophora	lycoperdoides				126	1
APA496255	Asterophora	parasitica				126	1
ABRR16S	Athelia	bombacina				126	1
BS09535	Basidiomycete	symbiont				126	1
AY662660	Boletellus	projectellus				126	1
AY657011	Boletellus	shichianus				126	1
AY662668	Boletinellus	merulioides				126	1
BSRGEA	Boletus	satanas				126	1
AY219404	Byssomerulius	albostramineu				126	1
AY752974	Callistosporium	sp				126	1
AY665773	Calostoma	cinnabarinum				126	1
AY445118	Campanella	subdendrophora				126	1
AB014401	Cephaliophora	irregularis				126	1
AB001112	Cephaliophora	tropica				126	1
AF518574	Ceraceomyces	serpens				126	1
AF334906	Ceriporiopsis	subvermispora				126	1
AY271801	Phlebia	sp				126	1
AY657010	Chlorophyllum	agaricoides				126	1
AF104340	Chorioactis	geaster				126	1
CV18SRRN	Chroogomphus	vinicolor				126	1
AY771609	Clitocybe	candicans				126	1
CCRRNA1	Coprinus	cinereus				126	1
AY665772	Coprinus	comatus				126	1
AF026633	Cortinarius	iodes				126	1
AF026617	Cyathus	striatus				126	1
AF516557	Datronia	mollis				126	1
AF104342	Donadinia	sp				126	1
AY657007	Entoloma	sinuatum				126	1
AY654890	Exidia	uvapsassa				126	1
AY654888	Fibricium	rude				126	1
AF004948	Galiella	rufa				126	1
AF518578	Galzinia	incrustans				126	1
AY705969	Ganoderma	tsugae				126	1
GAB560802	Gloeophyllum	abietinum				126	1
GSE540308	Gloeophyllum	sepiarium				126	1
GG18SRRN1	Gomphidius	oregonensis				126	1
AF334915	Heliocybe	sulcata				126	1
AY752965	Hygrocybe	conica				126	1
AY787215	Hypholoma	sublateritium				126	1
AB072228	Itersonilia	perplexans				126	1
AY654886	Laccaria	ochropurpurea				126	1
AY654885	Lacrymaria	velutina				126	1
AY705966	Laetiporus	sulphureus				126	1
AF516518	Lentinus	tigrinus				126	1
LPRRDA	Lepiota	procera				126	1
AF076385	Leucoagaricus	gongylophorus				126	1
AF426952	Limnoperdon	incarnatum				126	1
AF026619	Lycoperdon	sp				126	1
AY771602	Macrolepiota	dolichaula				126	1
AY787214	Marasmius	alliaceus				126	1
AF334921	Neolentiporus	maculatissimu				126	1
NGL534443	Neoplaconema	gloeosporioid				126	1
AF334754	Nia	vibrissa				126	1
AF334922	Oligoporus	rennyi				126	1
AF334923	Ossicaulis	lignatilis				126	1
AF006308	Otidea	onotica				126	1
PRU59086	Panus	rudis				126	1
PS18SRRN1	Paragyrodon	sphaerosporus				126	1
AF026628	Paxillus	panuoides				126	1
AY662662	Paxillus	vernalis				126	1
AY752970	Phaeomarasmius	proximans				126	1
PCU59084	Phanerochaete	chrysosporium				126	1
AY178022	Phellinus	bicuspidatus				126	1
AY219402	Phlebia	tremellosa				126	1
PR18SRRN	Phylloporus	rhodoxanthus				126	1
AY707090	Phyllotopsis	sp				126	1
AY293146	Physalacria	inflata				126	1
AF516524	Polyporus	arcularius				126	1
AF516528	Polyporus	brumalis				126	1
AF516565	Polyporus	guianensis				126	1
AF516567	Polyporus	leprieurii				126	1
AF516570	Polyporus	mikawai				126	1
AF516542	Polyporus	tricholoma				126	1
AF516599	Polyporus	tuberaster				126	1
AF516580	Polyporus	varius				126	1
AF516582	Polyporus	virgatus				126	1
AF334932	Postia	leucomallela				126	1
PN18SRRNA	Pseudoplectania	nigrella				126	1
AY219396	Rhizochaete	americana				126	1
AY219395	Rhizochaete	brunnea				126	1
AY219398	Rhizochaete	filamentosa				126	1
AY219399	Rhizochaete	radicata				126	1
RZSW1S	Rhizoctonia	zeae				126	1
AF104346	Sarcosoma	mexicanum				126	1
SC16RRNA	Schizophyllum	commune				126	1
AY705952	Schizophyllum	radiatum				126	1
AY220610	Scleroconidioma	sphagnicola				126	1
SHI437602	Serpula	himantioides				126	1
SLA437601	Serpula	lacrymans				126	1
AF334936	Sistotrema	musicola				126	1

Accession		18S	NSA3																							NSI1																				Speceis	Cluster
Number	Genus	Species	A	A	A	C	T	C	T	G	T	C	G	T	G	C	T	G	G	G	G	A	T	A	-	G	A	T	T	G	A	A	T	G	G	C	T	T	A	G	T	G	A	G	G	per cluster	number
AF305717	Sparassis	crispa																																												126	1
SSU59096	Sparassis	spathulata																																												126	1
AF006310	Strobiloscypha	keliae																																												126	1
AF026635	Stropharia	rugosoannulata																																												126	1
PA18SRRN	Tapinella	atrotomentosa																																												126	1
AF079066	Termitomyces	albuminosus																																												126	1
AF026627	Thelephora	sp																																												126	1
TSU59098	Trametes	suaveolens																																												126	1
AY309019	Trametes	versicolor																																												126	1
AY803753	Trechispora	sp																																												126	1
U63474	Trichaptum	abietinum																																												126	1
U63477	Trichaptum	laricinum																																												126	1
AY757267	Tricholoma	aestuans																																												126	1
AY293161	Tricholoma	inamoenum																																												126	1
TMU62538	Tricholoma	matsutake																																												126	1
AY654883	Tricholoma	saponaceum																																												126	1
AY445117	Tricholomataceae	sp																																												126	1
AF026625	Tulostoma	macrocephala																																												126	1
UHRRNA18S	U	hiemalis																																												126	1
AB072227	Udeniomyces	pannonicus																																												126	1
AF038589	Unidentified	ascomycota																																												126	1
AF104347	Urnula	craterium																																												126	1
XCRGEA	Xerocomus	chrysenteron																																												126	1
AF334904	Bjerkandera	adusta																																						N						126	1
DQU59067	Daedalea	quercina				N																																								126	1
FFU59069	Fomes	fomentarius																																						N						126	1
AY154701	Alternaria	dauci																																				C								108	2
AY154702	Alternaria	dianthi																																				C								108	2
AY154713	Alternaria	helianthi																																				C								108	2
AY154703	Alternaria	japonica																																				C								108	2
AY154684	Alternaria	longipes																																				C								108	2
AY154689	Alternaria	macrospora																																				C								108	2
AY154683	Alternaria	mali																																				C								108	2
AY154685	Alternaria	petroselini																																				C								108	2
AY154704	Alternaria	radicina																																				C								108	2
AR05199	Alternaria	raphani																																				C								108	2
AY154716	Alternaria	solani																																				C								108	2
AY154719	Alternaria	sp																																				C								108	2
AY154712	Alternaria	tenuissima																																				C								108	2
AY154695	Alternaria	triticina																																				C								108	2
AY154696	Alternaria	zinniae																																				C								108	2
APU549800	Amandinea	punctata																																				C								108	2
AF119501	Anamylopsora	pulcherrima																																				C								108	2
AY313953	Asteromassaria	olivaceohirt																																				C								108	2
BMU42656	Balsamia	magnata																																				C								108	2
AF054905	Balsamia	vulgaris																																				C								108	2
AY544696	Barssia	cf																																				C								108	2
BOU42657	Barssia	oregonensis																																				C								108	2
BAE549801	Buellia	aethalea																																				C								108	2
BEL421680	Buellia	elegans																																				C								108	2
AF184749	Bunodophoron	australe																																				C								108	2
AY220611	Capnobotryella	renispora																																				C								108	2
CSP301706	Capnobotryella	sp																																				C								108	2
AY646226	Cf	Phoma																																				C								108	2
AY342012	Chaetomella	raphigera																																				C								108	2
CSP301961	Chaetomella	sp																																				C								108	2
AF054904	Choiromyces	meandriformis																																				C								108	2
CVU42661	Choiromyces	venosus																																				C								108	2
AF184750	Cladia	aggregata																																				C								108	2
CD43464	Clathrospora	diplospora																																				C								108	2
U77668	Coccodinium	bartschii																																				C								108	2
CS42479	Cochliobolus	sativus																																				C								108	2
AY629206	Coniothyrium	olivaceum																																				C								108	2
CSP301702	Coniothyrium	sp																																				C								108	2
CB42481	Cucurbitaria	berberidis																																				C								108	2
CE42482	Cucurbitaria	elongata																																				C								108	2
DVU42659	Dingleya	verrucosa																																				C								108	2
AY342014	Discohainesia	oenotherae																																				C								108	2
AY544699	Helvella	cf																																				C								108	2
U53378	Helvella	lacunosa																																				C								108	2
AF046216	Helvella	terrestris																																				C								108	2
AF184754	Heterodea	muelleri																																				C								108	2
HBO421685	Heterodermia	boryi																																				C								108	2
HWY18700	Hortaea	werneckii																																				C								108	2
HAD549803	Hyperphyscia	adglutinata																																				C								108	2
AF053728	Kirschsteiniothelia	elatera																																				C								108	2
LVU42662	Labyrinthomyces	varius																																				C								108	2
U70959	Leifidium	tenerum																																				C								108	2
LD04205	Leptosphaeria	doliolum																																				C								108	2
LM04238	Leptosphaeria	maculans																																				C								108	2
LM04235	Leptosphaeria	microscopica																																				C								108	2
AY154718	Lewia	infectoria																																				C								108	2
AY584679	Lobaria	scrobiculata																																				C								108	2
MCRDNA18S	M	castaneae																																				C								108	2
AF184755	Metus	conglomeratus																																				C								108	2
MM43449	Mycosphaerella	mycopappi																																				C								108	2
AJ619957	Myrothecium	sp																																				C								108	2
AF117981	Neophyllis	melacarpa																																				C								108	2
AF250823	Paraphaeosphaeria	agavensis																																				C								108	2
AF250824	Paraphaeosphaeria	conglomer																																				C								108	2
AF250825	Paraphaeosphaeria	filamento																																				C								108	2
AF250819	Paraphaeosphaeria	glauco-pu																																				C								108	2
AF250817	Paraphaeosphaeria	michotii																																				C								108	2
AF250818	Paraphaeosphaeria	nolinae																																				C								108	2
AF250822	Paraphaeosphaeria	obtusispo																																				C								108	2
AF250826	Paraphaeosphaeria	quadrisep																																				C								108	2
AB096264	Paraphaeosphaeria	sp																																				C								108	2
AF450289	Phacopsis	huuskonenii																																				C								108	2
AF218792	Phoma	herbarum																																				C								108	2

Accession	18S		NSA3																							NSI1																				Speceis	Cluster
Number	Genus	Species	A	A	A	C	T	C	T	G	T	C	G	T	G	C	T	G	G	G	G	A	T	A	-	G	A	T	T	G	A	A	T	G	G	C	T	T	A	G	T	G	A	G	G	per cluster	number
AF218789	Phoma	sp																																			C								108	2
PAD421686	Physcia	adscendens																																			C								108	2
AY648101	Physcia	aipolia																																			C								108	2
AY648100	Physcia	caesia																																			C								108	2
PDI507615	Physcia	dimidiata																																			C								108	2
AY648102	Physcia	stellaris																																			C								108	2
AF184758	Pilophorus	strumaticus																																			C								108	2
AF006306	Pindara	terrestris																																			C								108	2
AF119502	Placopsis	gelida																																			C								108	2
PH43458	Pleospora	herbarum																																			C								108	2
AY548822	Protoblastenia	calva																																			C								108	2
AY425662	Protoblastenia	lilacina																																			C								108	2
AY548820	Protoblastenia	rupestris																																			C								108	2
PFA549807	Pyxine	farinosa																																			C								108	2
RDU42660	Reddellomyces	donkii																																			C								108	2
AY530887	Rhizoplaca	peltata																																			C								108	2
RGE549808	Rinodina	gennarii																																			C								108	2
SSO549809	Santessonia	sorediata																																			C								108	2
AY543588	Shiraia	bambusicola																																			C								108	2
AF117983	Sphaerophorus	globosus																																			C								108	2
AF140236	Stereocaulon	paschale																																			C								108	2
U70961	Stereocaulon	ramulosum																																			C								108	2
AF184761	Stereocaulon	vesuvianum																																			C								108	2
AY342013	Synchaetomella	lunatospora																																			C								108	2
TE18SR	T	excavatum																																			C								108	2
TSC549810	Tornabea	scutellifera																																			C								108	2
AF119499	Trapelia	involuta																																			C								108	2
AF119500	Trapelia	placodioides																																			C								108	2
AF054902	Tuber	borchii																																			C								108	2
TGU42663	Tuber	gibbosum																																			C								108	2
UAL301714	Ulocladium	alternariae																																			C								108	2
AF548106	Ulocladium	botrytis																																			C								108	2
AY744286	Uncultured	Leptosphaeriacea																																			C								108	2
UCU42658	Underwoodia	columnaris																																			C								108	2
WSU42655	Wynnella	silvicola																																			C								108	2
AB054676	Candida	castellii							C																																						52	3
AB013561	Candida	coipomoensis							C																																						52	3
AB013524	Candida	ergastensis							C																																						52	3
AB013521	Candida	fluviatilis							C																																						52	3
AY198398	Candida	glabrata							C																																						52	3
AB013519	Candida	glucosophila							C																																						52	3
AB054678	Candida	humilis							C																																						52	3
AB013551	Candida	membranifaciens							C																																						52	3
AB054677	Candida	milleri							C																																						52	3
AB016584	Candida	palmioleophila							C																																						52	3
AB013544	Candida	pseudoglaebosa							C																																						52	3
AB013523	Candida	saitoana							C																																						52	3
DH17SDNA	Debaryomyces	hansenii							C																																						52	3
EAS496251	Eremothecium	ashbyi							C																																						52	3
ECO496250	Eremothecium	coryli							C																																						52	3
AF113137	Eremothecium	gossypii							C																																						52	3
U53443	Holleya	sinecauda							C																																						52	3
KP18SRRN1	Kluyveromyces	phaffi							C																																						52	3
KT18SRRNA	Kluyveromyces	thermotolerants							C																																						52	3
KV18SRNA	Kluyveromyces	viticola							C																																						52	3
KW18SRRN1	Kluyveromyces	waltii							C																																						52	3
KA18SRRNA	Kluyveromyces	aestuarii							C																																						52	3
AB016510	Kluyveromyces	africanus							C																																						52	3
AY198400	Kluyveromyces	delphensis							C																																						52	3
KD18SRRNA	Kluyveromyces	dobzhanskii							C																																						52	3
AY325966	Kluyveromyces	hubeiensis							C																																						52	3
AB054673	Kluyveromyces	lactis							C																																						52	3
KL18SRRNA	Kluyveromyces	lodderae							C																																						52	3
AB054675	Kluyveromyces	marxianus							C																																						52	3
AB012264	Kluyveromyces	nonfermentans							C																																						52	3
AB087374	Kluyveromyces	sp							C																																						52	3
KW18SRRNA	Kluyveromyces	wickerhamii							C																																						52	3
AB054674	Kluyveromyces	yarrowii							C																																						52	3
AB013513	Pichia	farinosa							C																																						52	3
SCZ75577	Naumovia	castellii							C																																						52	3
SD18SRNA	Saccharomyces	dairensis							C																																						52	3
SPAR18S	Saccharomyces	paradoxus							C																																						52	3
SPAS18S	Saccharomyces	pastorianus							C																																						52	3
AY046227	Saccharomyces	bayanus							C																																						52	3
SCA271813	Saccharomyces	cariocanus							C																																						52	3
AY218892	Saccharomyces	cerevisiae							C																																						52	3
AY251641	Saccharomyces	dairenensis							C																																						52	3
AY046235	Saccharomyces	kunashirensis							C																																						52	3
AY046231	Saccharomyces	martiniae							C																																						52	3
SMI271812	Saccharomyces	mikatae							C																																						52	3
AB016512	Saccharomyces	naganishii							C																																						52	3
AY251643	Saccharomyces	servazzii							C																																						52	3
AY251627	Saccharomyces	sp							C																																						52	3
AF026621	Scleroderma	citrina							C																																						52	3
TDSRSR	Torulaspora	delbrueckii							C																																						52	3
AB087380	Torulaspora	sp							C																																						52	3
AY227015	Zygosaccharomyces	rouxii							C																																						52	3
BDDA	Blastomyces	dermatitidis					C																														C								37	4
BNRR18S	Byssochlamys	nivea					C																														C								37	4
AY177297	Chlamydosauromyces	punctatu					C																														C								37	4
CIDA	Coccidiodes	immitis					C																														C								37	4
DCA421684	Diploicia	canescens					C																														C								37	4
AB031391	Eladia	saccula					C																														C								37	4
ELU45441	Elaphomyces	leveillei					C																														C								37	4
EJ21298	Eupenicillium	javanicum					C																														C								37	4
HANRRLB	Hamigera	avellanea					C																														C								37	4
MICBSD	Merimbla	ingelheimensis					C																														C								37	4
OEU45442	Onygena	equina					C																														C								37	4

Accession	18S		NSA3																							NSI1																				Speceis	Cluster
Number	Genus	Species	A	A	A	C	T	C	T	G	T	C	G	T	G	C	T	G	G	G	G	A	T	A	-	G	A	T	T	G	A	A	T	G	G	C	T	T	A	G	T	G	A	G	G	per cluster	number
AF238302	Paracoccidioides	brasiliens					C																															C								37	4
AB003946	Penicilliopsis	clavariiform					C																															C								37	4
AB033534	Penicillium	argillaceum					C																															C								37	4
AF245228	Penicillium	crateriforme					C																															C								37	4
AF245234	Penicillium	diversum					C																															C								37	4
AF245267	Penicillium	funiculosum					C																															C								37	4
AF245270	Penicillium	glabrum					C																															C								37	4
AB032070	Penicillium	lagena					C																															C								37	4
AF245240	Penicillium	minioluteum					C																															C								37	4
D88319	Penicillium	namyslowskii					C																															C								37	4
AF245235	Penicillium	phialosporum					C																															C								37	4
AF245239	Penicillium	pinophilum					C																															C								37	4
AF245242	Penicillium	pittii					C																															C								37	4
AF245249	Penicillium	purpurogenum					C																															C								37	4
AY256855	Penicillium	radicum					C																															C								37	4
AF245232	Penicillium	rugulosum					C																															C								37	4
AB027410	Penicillium	sacculum					C																															C								37	4
AF245245	Penicillium	siamense					C																															C								37	4
AF245254	Penicillium	sp					C																															C								37	4
AF510496	Penicillium	verruculosum					C																															C								37	4
RCO421691	Rinodinella	controversa					C																															C								37	4
TBIFOE	Talaromyces	bacillisporus					C																															C								37	4
AB003947	Talaromyces	luteus					C																															C								37	4
TCRR18S	Thermoascus	crustaceus					C																															C								37	4
AB003944	Trichocoma	paradoxa					C																															C								37	4
AB002081	Warcupiella	spinulosa					C																															C								37	4
AB022928	Bullera	boninensis							A																																			A	32	5
BCSSRRGA	Bullera	crocea							A																																			A	32	5
AB118873	Bullera	formosana							A																																			A	32	5
AB118872	Bullera	melastomae							A																																			A	32	5
AF314993	Bullera	mrakii							A																																			A	32	5
AF314994	Bullera	pseudohuiaensis							A																																			A	32	5
AY313029	Bullera	sp							A																																			A	32	5
AB022929	Bullera	waltii							A																																			A	32	5
AB032614	Cryptococcus	aerius							A																																			A	32	5
AB032627	Cryptococcus	dimennae							A																																			A	32	5
AB032629	Cryptococcus	flavus							A																																			A	32	5
AB032631	Cryptococcus	fuscescens							A																																			A	32	5
AB032633	Cryptococcus	gastricus							A																																			A	32	5
AB032634	Cryptococcus	gilvescens							A																																			A	32	5
CNDA	Cryptococcus	neoformans							A																																			A	32	5
AB100440	Cryptococcus	surugaensis							A																																			A	32	5
AB032649	Cryptococcus	terreus							A																																			A	32	5
AB049615	Dioszegia	aurantiaca							A																																			A	32	5
AB032638	Dioszegia	hungarica							A																																			A	32	5
AY313036	Dioszegia	sp							A																																			A	32	5
AF385443	Dioszegia	zsoltii							A																																			A	32	5
AF356652	Filobasidiella	neoformans							A																																			A	32	5
AB075544	Filobasidium	capsuligenum							A																																			A	32	5
CHU75449	Camptobasidium	hydrophilum														A																													30	6
AY707092	Leucosporidium	scottii														A																													30	6
RG18SRRA	R	glutinis														A																													30	6
AB073269	Rhodosporidium	azoricum														A																													30	6
AB073270	Rhodosporidium	babjevae														A																													30	6
AB073271	Rhodosporidium	diobovatum														A																													30	6
RFU77395	Rhodosporidium	fluviale														A																													30	6
AB073273	Rhodosporidium	kratochvilov														A																													30	6
AB073274	Rhodosporidium	lusitaniae														A																													30	6
AB073275	Rhodosporidium	sphaerocarpu														A																													30	6
RGSRSRNA	Rhodosporidium	toruloides														A																													30	6
AB021869	Rhodotorula	glutinis														A																													30	6
RG18SRRNA	Rhodotorula	graminis														A																													30	6
AY657013	Rhodotorula	hordea														A																													30	6
AB042787	Rhodotorula	mucilaginosa														A																													30	6
AF331945	Rhodotorula	sp														A																													30	6
AB032658	Rhodotorula	yarrowii														A																													30	6
SRSRSRNA	Sporobolomyces	roseus														A																													30	6
AB021672	Sporidiobolus	johnsonii														A																													30	6
AB021694	Sporidiobolus	pararoseus														A																													30	6
AB021696	Sporidiobolus	ruineniae														A																													30	6
AB021697	Sporidiobolus	salmonicolor														A																													30	6
AY123321	Sporidiobolus	sp														A																													30	6
AB030321	Sporobolomyces	blumeae														A																													30	6
AB021670	Sporobolomyces	falcatus														A																													30	6
D66884	Sporobolomyces	griseoflavus														A																													30	6
AB030319	Sporobolomyces	nylandii														A																													30	6
AB030320	Sporobolomyces	poonsookiae														A																													30	6
AF331940	Sporobolomyces	sp														A																													30	6
ZEU77063	Zymoxenogloea	eriophori														A																													30	6
AB002065	Aspergillus	candidus					C																															C	G							23	7
AB003808	Aspergillus	cervinus					C																															C	G							23	7
AB008398	Aspergillus	clavatus					C																															C	G							23	7
AF18SRR2	Aspergillus	flavus					C																															C	G							23	7
AF548063	Aspergillus	fumigatus					C																															C	G							23	7
AF548065	Aspergillus	ochraceus					C																															C	G							23	7
AO18SRR4	Aspergillus	oryzae					C																															C	G							23	7
AB002078	Aspergillus	penicillioides					C																															C	G							23	7
AB002083	Aspergillus	proliferans					C																															C	G							23	7
AB002079	Aspergillus	restrictus					C																															C	G							23	7
AB179824	Aspergillus	sp					C																															C	G							23	7
AB106338	Aspergillus	tamarii					C																															C	G							23	7
AB002072	Aspergillus	ustus					C																															C	G							23	7
AB002064	Aspergillus	versicolor					C																															C	G							23	7
AB002082	Edyuillia	athecia					C																															C	G							23	7
AB002076	Eurotium	amstelodami					C																															C	G							23	7
AB002073	Eurotium	cristatum					C																															C	G							23	7
AB002084	Eurotium	repens					C																															C	G							23	7
ER970	Eurotium	rubrum					C																															C	G							23	7
AB002075	Hemicarpenteles	acanthospor					C																															C	G							23	7

Accession	18S		NSA3																							NSI1																				Speceis	Cluster
Number	Genus	Species	A	A	A	C	T	C	T	G	T	C	G	T	G	C	T	G	G	G	G	A	T	A	-	G	A	T	T	G	A	A	T	G	G	C	T	T	A	G	T	G	A	G	G	per cluster	number
AB002080	Hemicarpenteles	paradoxus					C																															C	G							23	7
NF21299	Neosartorya	fischeri					C																															C	G							23	7
AB002071	Petromyces	alliaceus					C																															C	G							23	7
DMU42651	Discina	macrospora																																		G		C								11	8
DVU42643	Disciotis	venosa																																		G		C								11	8
FSU42646	Fischerula	subcaulis																																		G		C								11	8
GEU42648	Gyromitra	esculenta																																		G		C								11	8
GMU42653	Gyromitra	melaleucoides																																		G		C								11	8
GMU42652	Gyromitra	montana																																		G		C								11	8
HCU42649	Hydnotrya	cerebriformis																																		G		C								11	8
LCU42647	Leucangium	carthusianum																																		G		C								11	8
PCU42650	Pseudorhizina	californica																																		G		C								11	8
VBU42645	Verpa	bohemica																																		G		C								11	8
VCU42644	Verpa	conica																																		G		C								11	8
AF334901	Amylostereum	laevigatum																																										A	9	9
CMA540306	Coniophora	marmorata																																										A	9	9
AF334912	Dichostereum	pallescens																																										A	9	9
DEX540307	Donkioporia	expansa																																										A	9	9
AY752971	Hydnellum	geogenium																																										A	9	9
AF026626	Hydnellum	sp																																										A	9	9
PNU59085	Peniophora	nuda																																										A	9	9
AF516601	Pseudofavolus	cucullatus																																										A	9	9
AF334938	Tyromyces	chioneus																																										A	9	9
AF026639	Clavariadelphus	pistillaris																														C													7	10
AF026620	Geastrum	saccatum																														C													7	10
AY293138	Kavinia	himantia																														C													7	10
MGU59082	Meripilus	giganteus																														C													7	10
AF026618	Sphaerobolus	stellatus																														C													7	10
SC18SRRN	Suillus	cavipes																														C													7	10
SSS18SRRN	Suillus	sinuspaulianus																														C													7	10
TAL495834	Taphrina	alni							C							A																												A	5	11
TEP495823	Taphrina	epiphylla							C							A																												A	5	11
TRO495830	Taphrina	robinsoniana							C							A																												A	5	11
TSA495820	Taphrina	sadebeckii							C							A																												A	5	11
TTO496252	Taphrina	tosquinetii							C							A																												A	5	11
AF026640	Clavulina	cristata						T																																					4	12
AY662663	Hygrophoropsis	aurantiaca						T																																					4	12
TCV12MO	Rhizoctonia	solani						T																																					4	12
RSSN2R	Rhizoctonia	sp						T																																					4	12
ATR243420	Acaulospora	trappei						T	C	A																																		A	4	13
OBY12830	Olpidium	brassicae						T	C	A																																		A	4	13
AY584734	Olpidium	bornovanus						T	C	A																																		A	4	13
AY744285	Uncultured	mycorrhiza						T	C	A																																		A	4	13
AY357266	Anguillospora	rubescens						G																														C								3	14
GLA301970	Glomerella	lagenaria						G																														C								3	14
MPRRDA	Mycosphaerella	punctiformis						G																														C								3	14
AF026622	Calvatia	gigantea					C																																						3	15
AF334910	Dendrocorticium	roseocarneu					C																																						3	15
AF518594	Vuilleminia	comedens					C																																						3	15
U70960	Pilophorus	acicularis							G																													C								3	16
AF184756	Pilophorus	cereolus							G																													C								3	16
AF184757	Pilophorus	robustus							G																													C								3	16
AB118874	Bullera	begoniae							A																											T								A	3	17
AY188386	Bullera	panici							A																											T								A	3	17
AY188389	Bullera	siamensis							A																											T								A	3	17
AB035897	Bannoa	hahajimana							G							A																													3	18
AB035720	Bannoa	syzygii							G							A																													3	18
AB178481	Sporobolomyces	gracilis							G							A																													3	18
AY635833	Glomus	mosseae						T	C	A						A																												A	3	19
AB041345	Scutellospora	cerradensis						T	C	A						A																												A	3	19
AY635832	Scutellospora	heterogama						T	C	A						A																												A	3	19
AF218791	Alternaria	alternata																																				C								2	20
AY154706	Alternaria	arborescens																																				C								2	20
AY154700	Alternaria	cinerariae																																				C								2	21
AY154705	Alternaria	citri																																				C								2	21
BASRSRNA	Bulleromyces	albus							A																														G					A	2	22
AB085797	Cryptococcus	flavescens							A																														G					A	2	22
AF453291	Bullera	anomala							A																																			A	2	23
BAD323	Bullera	armeniaca							A																																			A	2	23
AF453290	Bullera	pseudovariabilis							A							A																				T								A	2	24
BVSSRRGG	Bullera	variabilis							A							A																				T								A	2	24
AY705954	Dacrymyces	sp						T	C																																				2	25
AY771603	Dacryopinax	spathularia						T	C																																				2	25
AY546682	Monoblepharella	sp						T	C	A																																			2	26
AY635839	Oedogoniomyces	sp						T	C	A																																			2	26
AF368504	Basidiobolus	haptosporus				T		T	C	A														T																				A	2	27
AY635841	Basidiobolus	ranarum				T		T	C	A														T																				A	2	27
AGE012201	Archaeospora	leptoticha						T	C	A																						C												A	2	28
AB047309	Glomus	leptotichum						T	C	A																						C												A	2	28
AY662661	Strobilomyces	floccopus																										C				C													1	29
AF393043	Gautieria	otthii					C																									C													1	30
RUU42664	Rhizina	undulata																																			C	C								1	31
AY204586	Alatospora	acuminata																																				C								1	32
AF212309	Embellisia	sp																												G								C								1	33
AB05197	Alternaria	brassicicola																																				C								1	34
AF292092	Unguiculariopsis	damingshan																									-											C								1	35
AF292089	Cordierites	sprucei				G																																C								1	36
AY219403	Ceriporiopsis	gilvescens						G																																					1	37
AF010506	Monilinia	laxa																																				A								1	38
AB002066	Aspergillus	sparsus					C																															C	C							1	39
AB002063	Aspergillus	wentii					C																																G							1	40
AY124493	Auxarthron	zuffianum					C																					C										C								1	41
AB003952	Chromocleista	cinnabarina					C																															C						A		1	42
AF245238	Penicillium	variabile					C																															C						R		1	43
BMSSRRGD	Bullera	miyagiana							G																																				1	44
AF334939	Vararia	insolita							C																																			A	1	45
AY150055	Rhodotorula	minuta				T			G							A																													1	46
HCU17504	Hyaloraphidium	curvatum				G		T								A																													1	47
AY771610	Calocera	cornea						T	C																																				1	48

Accession		18S	NSA3																							NSI1																				Speceis	Cluster
Number	Genus	Species	A	A	A	C	T	C	T	G	T	C	G	T	G	C	T	G	G	G	G	A	T	A	-	G	A	T	T	G	A	A	T	G	G	C	T	T	A	G	T	G	A	G	G	per cluster	number
AY662659	Suillus	pictus						T	C																								C													1	49
MAL271630	Mortierella	alpina						T	G	A																																				1	50
AY635828	Mortierella	sp						T	G	A																							C													1	51
AF108495	Verticillium	rexianum						T	G	A																																				1	52
AY635838	Rozella	allomycis				T		T	C	A																																				1	53
AB013526	Candida	atmosphaerica							C																																					1	54
KP18SRRA	Kluyveromyces	polysporus							C		G																																			1	55
AB018174	Candida	silvanorum							C																											T			G							1	56
AF234840	Medeolaria	farlowii																																		T	C	C	G					A		1	57
AF113506	Chaetosphaeridium	globosum				-																																								126	58
AA18SRNA	A	agrestis				T		T	C	A						A																				T	C	C	G					A		51	59
AURRNA18S	A	undulatum				T		T	C	A						A																				T	C	C	G					A		51	59
AY126951	Alophosia	azorica				T		T	C	A						A																				T	C	C	G					A		51	59
ANI243169	Andreaea	nivalis				T		T	C	A						A																				T	C	C	G					A		51	59
AMA275005	Andreaeobryum	macrosporum				T		T	C	A						A																				T	C	C	G					A		51	59
APY16015	Anthoceros	punctatus				T		T	C	A						A																				T	C	C	G					A		51	59
AY126952	Atrichum	androgynum				T		T	C	A						A																				T	C	C	G					A		51	59
AY126953	Atrichum	oerstedianum				T		T	C	A						A																				T	C	C	G					A		51	59
AY126954	Bartramiopsis	lescurii				T		T	C	A						A																				T	C	C	G					A		51	59
AY126949	Buxbaumia	piperi				T		T	C	A						A																				T	C	C	G					A		51	59
CA18SRR	C	arguta				T		T	C	A						A																				T	C	C	G					A		51	59
AY126955	Dawsonia	longifolia				T		T	C	A						A																				T	C	C	G					A		51	59
AY126956	Dawsonia	polytrichoides				T		T	C	A						A																				T	C	C	G					A		51	59
DDE16519	Dendroligotrichum	dendroides				T		T	C	A						A																				T	C	C	G					A		51	59
DFO275008	Diphyscium	foliosum				T		T	C	A						A																				T	C	C	G					A		51	59
FP18SRNA	F	pusilla				T		T	C	A						A																				T	C	C	G					A		51	59
AY126958	Hebantia	rigida				T		T	C	A						A																				T	C	C	G					A		51	59
HROY18972	Hypopterygium	didictyon				T		T	C	A						A																				T	C	C	G					A		51	59
AY126959	Itatiella	ulei				T		T	C	A						A																				T	C	C	G					A		51	59
JA18SRR	J	autumnalis				T		T	C	A						A																				T	C	C	G					A		51	59
JL18SRRNA	J	leiantha				T		T	C	A						A																				T	C	C	G					A		51	59
LH18SRRNA	L	heterophylla				T		T	C	A						A																				T	C	C	G					A		51	59
AF208403	Lyellia	aspera				T		T	C	A						A																				T	C	C	G					A		51	59
AY126960	Meiotrichum	lyallii				T		T	C	A						A																				T	C	C	G					A		51	59
AF228668	Oedipodium	griffithianum				T		T	C	A						A																				T	C	C	G					A		51	59
AY126961	Oligotrichum	austroaligerum				T		T	C	A						A																				T	C	C	G					A		51	59
AY126962	Oligotrichum	hercynicum				T		T	C	A						A																				T	C	C	G					A		51	59
AY126963	Oligotrichum	parallelum				T		T	C	A						A																				T	C	C	G					A		51	59
PA18RRNA	P	adiantoides				T		T	C	A						A																				T	C	C	G					A		51	59
PE18SRRNA	P	epiphylla				T		T	C	A						A																				T	C	C	G					A		51	59
AF223017	Physcomitrium	lorentzii				T		T	C	A						A																				T	C	C	G					A		51	59
AY126966	Pogonatum	cirratum				T		T	C	A						A																				T	C	C	G					A		51	59
AY126967	Pogonatum	contortum				T		T	C	A						A																				T	C	C	G					A		51	59
AY126968	Pogonatum	dentatum				T		T	C	A						A																				T	C	C	G					A		51	59
AY126969	Pogonatum	japonicum				T		T	C	A						A																				T	C	C	G					A		51	59
AY126970	Pogonatum	microstomum				T		T	C	A						A																				T	C	C	G					A		51	59
AY126974	Pogonatum	spinulosum				T		T	C	A						A																				T	C	C	G					A		51	59
AF208406	Pogonatum	urnigerum				T		T	C	A						A																				T	C	C	G					A		51	59
AY126976	Polytrichadelphus	pseudopoly				T		T	C	A						A																				T	C	C	G					A		51	59
AY126978	Polytrichastrum	longisetum				T		T	C	A						A																				T	C	C	G					A		51	59
AY126979	Polytrichum	brachymitrium				T		T	C	A						A																				T	C	C	G					A		51	59
AY126980	Polytrichum	juniperinum				T		T	C	A						A																				T	C	C	G					A		51	59
AY126981	Polytrichum	piliferum				T		T	C	A						A																				T	C	C	G					A		51	59
AY126982	Polytrichum	subpilosum				T		T	C	A						A																				T	C	C	G					A		51	59
AY126983	Psilopilum	laevigatum				T		T	C	A						A																				T	C	C	G					A		51	59
SB18SRRNA	S	brongartii				T		T	C	A						A																				T	C	C	G					A		51	59
AY126984	Steereobryon	subulirostrum				T		T	C	A						A																				T	C	C	G					A		51	59
TT18SRRNA	T	tomentella				T		T	C	A						A																				T	C	C	G					A		51	59
TLE269686	Takakia	lepidozioides				T		T	C	A						A																				T	C	C	G					A		51	59
AF023691	Tetraplodon	mnioides				T		T	C	A						A																				T	C	C	G					A		51	59
TLA239055	Treubia	lacunosa				T		T	C	A						A																				T	C	C	G					A		51	59
AY206425	Caulerpa	cupressoides							A																																				A	32	60
AY206426	Caulerpa	webbiana							A																																				A	32	60
AF479758	Chlorella	sp							A																																				A	32	60
AY497565	Liagora	boergesenii							A																																				A	32	60
AY497569	Liagora	decussata							A																																				A	32	60
AY497570	Liagora	orientalis							A																																				A	32	60
AY497573	Liagora	segawae							A																																				A	32	60
AY497576	Liagora	valida							A																																				A	32	60
AY497577	Yamadaella	caenomyce							A																																				A	32	60
AB021684	Marchantia	polymorpha				A		T	C	A						A																				T	C	C	G					A		3	61
PQ18SRRNA	P	quadrata				A		T	C	A						A																				T	C	C	G					A		3	61
RH18SRRN	Reboulia	hemisphaerica				A		T	C	A						A																				T	C	C	G					A		3	61
AF141969	Cepedea	virguloidea				T		T	C	A																										T									C	2	62
AF141970	Opalina	ranarum				T		T	C	A																										T									C	2	62
PSA16S	Pyrenomonas	salina				A		T	C	A						A																				T	C	C	G					A	A	2	63
RSP7286	Rhodomonas	sp				A		T	C	A						A																				T	C	C	G					A	A	2	63
CP18SNU	Guillardia	theta				G		T	C	A						A																				T	C	C	G					A	A	2	64
TAM7287	Teleaulax	amphioxeia				G		T	C	A						A																				T	C	C	G					A	A	2	64
BAP17603	Buxbaumia	aphylla				T		T	C	A						A						G														T	C	C	G					A		2	65
DSU16520	Dawsonia	superba				T		T	C	A						A						G														T	C	C	G					A		2	65
AY497563	Ganonema	farinosum							A																							G													A	1	66
AY497564	Helminthocladia	australis							A																																		G		A	1	67
AY497567	Liagora	ceranoides							A																									T											A	1	68
AY360454	Goniomonas	sp			C	T		T	C	A						A																														1	69
UCR536451	Uncultured	cryptomonad				T		T	C	A						A		R																											A	1	70
GW16RRNA	Gloeochaete	wittrockiana						T	C	A						A																			A			C	G					A	C	1	71
FHRRNA	Funaria	hygrometrica				T		T	C	A						A																				T	C	C	G							1	72
GNL16SLA	Glaucocystis	nostochinearum				T		T	C	A						A																				T	C	C	G					A	A	1	73
PSU7285	Proteomonas	sulcata						T	C	A						A																				T	C	C	G					A	A	1	74

Accession Number	Genus	5.8S Species	58A2R / 58A1F																Species per cluster	Cluster number
			58A2R	3'	C	G	A	T	G	A	A	G	A	A	C	G	C			
			58A1F				A	T	G	A	A	G	A	A	C	G	C	A	G 3'	
AF165922	Glomus	brasilianum																	460	1
AY373922	Penicillium	lividum																	460	1
AF374717	Pisolithus	tinctorius																	460	1
AF444442	Cryptococcus	cellulolyticus																	460	1
RSRRITSA	Rhizopogon	subcaerulescens																	460	1
CGE510274	Cenococcum	geophilum													-				22	2
AY826767	Septoria	glycines													-				22	2
AF270781	Pisolithus	sp											G						2	3
CFR486964	Calvatia	fragilis				C													1	4
AF097575	Elsinoe	leucospermi												-					1	5
AF333489	Phakopsora	pachyrhizi														A			1	6
AF096976	Pisolithus	arhizus										A						N	1	7
RSRRITSA	Rhizopogon	subcaerulescens																	1	8
AF401156	Acer	macrophyllum															T		75	9
AF041353	Pseudotsuga	menziesii															T		75	9
BFO491208	Begonia	formosana																	68	10
AB162910	Chlorella	vulgaris																	68	10
AF401149	Acer	decandrum															T		C	1	11

5.8S

Accession Number	Genus	Species	58A2R 3' C G A T G A A G A A C G C<br>58A1F A T G A A G A A C G C A G 3'	Species per cluster	Cluster number
AY484673	Agaricus	abruptibulbus		460	1
AY484675	Agaricus	albolutescens		460	1
AY484690	Agaricus	arvensis		460	1
AY484671	Agaricus	augustus		460	1
AY484678	Agaricus	bernardii		460	1
AY484697	Agaricus	blazei		460	1
AY484682	Agaricus	excellens		460	1
AY484683	Agaricus	fissuratus		460	1
AY484677	Agaricus	fuscovelatus		460	1
AY484685	Agaricus	hondensis		460	1
AY484686	Agaricus	macrocarpus		460	1
AY484670	Agaricus	nivescens		460	1
AY484689	Agaricus	xanthodermus		460	1
AF124713	Agrocybe	praecox		460	1
ASP419181	Alpova	sp		460	1
AF074920	Alpova	trappei		460	1
AY550243	Amanita	bisporigera		460	1
AY213562	Armillaria	calvescens		460	1
AY213583	Armillaria	cepistipes		460	1
AY213571	Armillaria	gallica		460	1
AY213558	Armillaria	gemina		460	1
AY213587	Armillaria	mellea		460	1
AY213574	Armillaria	nabsnona		460	1
AY213554	Armillaria	ostoyae		460	1
AY213567	Armillaria	sinapina		460	1
AY213580	Armillaria	sp		460	1
AY213590	Armillaria	tabescens		460	1
AF454424	Artomyces	colensoi		460	1
AF454415	Artomyces	enigmatus		460	1
AF360740	Ascomycete	CBS		460	1
AY243054	Ascomycete	sp		460	1
AY373845	Aspergillus	cervinus		460	1
AF459727	Aspergillus	clavatonanicus		460	1
AY373847	Aspergillus	clavatus		460	1
AY373851	Aspergillus	fumigatus		460	1
AF400603	Aspergillus	longivesica		460	1
AF400606	Aspergillus	pallidus		460	1
AF400612	Aspergillus	rhizopodus		460	1
AY139394	Aureobasidium	pullulans		460	1
GRO430208	Axenic	ericoid		460	1
AY233339	Bensingtonia	changbaiensis		460	1
AB040114	Bensingtonia	thailandica		460	1
BRO419192	Boletinellus	rompelii		460	1
AY680962	Boletus	aereus		460	1
BER419188	Boletus	erythropus		460	1
BFR419186	Boletus	fragrans		460	1
BLU296294	Boletus	lupinus		460	1
AF335451	Boletus	mirabilis		460	1
BRH419189	Boletus	rhodoxanthus		460	1
AY750158	Boletus	zelleri		460	1
BPO237613	Bovista	polymorpha		460	1
AF453289	Bullera	anomala		460	1
AY487563	Bullera	cylindrica		460	1
AF444405	Bullera	derxii		460	1
AB118873	Bullera	formosana		460	1
AB118872	Bullera	melastomae		460	1
AF444399	Bullera	pseudoalba		460	1
AF444665	Bullera	sinensis		460	1
AY313029	Bullera	sp		460	1
AF444667	Bulleromyces	sp		460	1
AF357036	Calocybe	constricta		460	1
CCY486873	Calvatia	cyathiformis		460	1
CARRB	Candida	albicans		460	1
AY198398	Candida	glabrata		460	1

Accession		5.8S	58A2R	3' C G A T G A A G A A C G C		Species	Cluster
Number	Genus	Species	58A1F	A T G A A G A A C G C A G	3'	per cluster	number
AY220611	Capnobotryella	renispora				460	1
AY078151	Phialocephala	fortinii				460	1
AY372288	Xerocomus	sp				460	1
AY429053	Chaetomium	globosum				460	1
AF335457	Chalciporus	piperatus				460	1
AF205660	Chroogomphus	jamaicensis				460	1
AF205649	Chroogomphus	rutilus				460	1
CVITS	Chroogomphus	vinicolor				460	1
U66431	Clitocybe	lateritia				460	1
AF274375	Collybia	racemosa				460	1
AY720707	Coniothyrium	leucospermi				460	1
AY033105	Cortinarius	achrous				460	1
AF325581	Cortinarius	anomalus				460	1
AY174807	Cortinarius	anserinus				460	1
AF539710	Cortinarius	aurantiorufus				460	1
AY174801	Cortinarius	balteatocumatil				460	1
AF389169	Cortinarius	bolaris				460	1
AY174861	Cortinarius	boudieri				460	1
AF539715	Cortinarius	caelicolor				460	1
CCU56024	Cortinarius	caninus				460	1
AY174786	Cortinarius	cephalixus				460	1
AF325607	Cortinarius	citriolens				460	1
AY174794	Cortinarius	coalescens				460	1
AF389134	Cortinarius	coerulescens				460	1
AY033115	Cortinarius	collariatus				460	1
AF325580	Cortinarius	delibutus				460	1
AY174813	Cortinarius	dionysae				460	1
AY033113	Cortinarius	dulciolens				460	1
AF539725	Cortinarius	elaphinus				460	1
AF539716	Cortinarius	flammuloides				460	1
AF389166	Cortinarius	flavifolius				460	1
AF325605	Cortinarius	fraudulosus				460	1
AF539720	Cortinarius	icterinus				460	1
AF389133	Cortinarius	iodes				460	1
AF112144	Cortinarius	krombholzii				460	1
AF539718	Cortinarius	lignyotus				460	1
AF539734	Cortinarius	lividus				460	1
AY174853	Cortinarius	lustratus				460	1
AF539719	Cortinarius	magellanicus				460	1
COB238035	Cortinarius	obtusus				460	1
AF389164	Cortinarius	orellanus				460	1
AF389142	Cortinarius	pluvius				460	1
AY174854	Cortinarius	porphyropus				460	1
AY174804	Cortinarius	praestans				460	1
AY174864	Cortinarius	pseudonapus				460	1
AF539714	Cortinarius	punctatisporus				460	1
AF539724	Cortinarius	rapaceus				460	1
AF389127	Cortinarius	rotundisporus				460	1
AF539726	Cortinarius	rubrobasalis				460	1
AF325606	Cortinarius	sp				460	1
AF539729	Cortinarius	squamiger				460	1
AF389151	Cortinarius	teraturgus				460	1
AY174798	Cortinarius	triumphans				460	1
AY174796	Cortinarius	variicolor				460	1
AY174791	Cortinarius	variiformis				460	1
AY174790	Cortinarius	varius				460	1
AY174811	Cortinarius	vulpinus				460	1
AB085802	Cryptococcus	aureus				460	1
AF444442	Cryptococcus	cellulolyticus				460	1
AB035588	Cryptococcus	fragicola				460	1
AJ876527	Cryptococcus	laurentii				460	1
AY301024	Cryptococcus	luteolus				460	1
CPO581036	Cryptococcus	podzolicus				460	1
AY686645	Cryptococcus	sp				460	1

Accession Number	Genus	5.8S Species	58A2R<br>58A1F	3' C G A T G A A G A A C G C<br>A T G A A G A A C G C A G 3'	Species per cluster	Cluster number
AB100440	Cryptococcus	surugaensis			460	1
AF430260	Dermocybe	cf			460	1
DCU56039	Dermocybe	cinnamomea			460	1
DCU56040	Dermocybe	cinnamomeolutea			460	1
DFU56042	Dermocybe	fervida			460	1
DMU56045	Dermocybe	malicoria			460	1
DMU56046	Dermocybe	mallochii			460	1
DPU56054	Dermocybe	phoenicea			460	1
DSU56060	Dermocybe	sanguinea			460	1
DSU56065	Dermocybe	semisanguinea			460	1
AF323112	Dermocybe	sp			460	1
AF325583	Dermocybe	splendida			460	1
DSU56069	Dermocybe	subcinnabarina			460	1
AY313036	Dioszegia	sp			460	1
AF027763	Dothidea	hippophaeos			460	1
AF027764	Dothidea	insculpta			460	1
DCA244243	Dothiora	cannabinae			460	1
DEU244244	Dothiora	europaea			460	1
DRH244245	Dothiora	rhamni-alpinae			460	1
AY509551	Ductifera	sucina			460	1
UEC410868	Ectomycorrhizal	isolate			460	1
AF477002	Ectomycorrhizal	root			460	1
AY604527	Endoconidioma	populi			460	1
AY243615	Endoptychum	agaricoides			460	1
AY268220	Epacris	microphylla			460	1
AF072291	Ericoid	mycorrhizal			460	1
AF033435	Eupenicillium	brefeldianum			460	1
AF033414	Eupenicillium	cinnamopurpur			460	1
AF033432	Eupenicillium	ehrlichii			460	1
AF033418	Eupenicillium	hirayamae			460	1
EJ18358	Eupenicillium	javanicum			460	1
AF033409	Eupenicillium	lapidosum			460	1
AF033436	Eupenicillium	levitum			460	1
AF033460	Eupenicillium	parvum			460	1
AF033411	Eupenicillium	pinetorum			460	1
AF033437	Eupenicillium	reticulisporu			460	1
AF033444	Eupenicillium	stolkiae			460	1
AJ608670	Fellomyces	sp			460	1
AF356652	Filobasidiella	neoformans			460	1
AY566889	Foliar	endophyte			460	1
AY546007	Fungal	endophyte			460	1
AF437775	Fungal	isolate			460	1
AY843062	Fungal	sp			460	1
AF178397	Fusarium	ambrosium			460	1
AF111061	Fusarium	flocciferum			460	1
AY667491	Fusarium	oxysporum			460	1
AY569560	Fusarium	solani			460	1
AY433805	Fusarium	sp			460	1
AJ585442	Galerina	arctica			460	1
AJ585480	Galerina	atkinsoniana			460	1
AJ585495	Galerina	badipes			460	1
AJ585506	Galerina	chionophila			460	1
AY281021	Galerina	clavata			460	1
AJ585440	Galerina	laevis			460	1
AY228347	Galerina	marginata			460	1
AJ585490	Galerina	nana			460	1
AJ585477	Galerina	pumila			460	1
AJ585475	Galerina	stylifera			460	1
AF165922	Glomus	brasilianum			460	1
GOU81986	Glomus	occultum			460	1
AY174700	Glomus	sp			460	1
AY077472	Gomphidius	glutinosus			460	1
AY077474	Gomphidius	nigricans			460	1
AY256709	Gymnopus	luxurians			460	1

Accession Number	Genus	5.8S Species	58A2R / 58A1F	3' C G A T G A A G A A C G C / A T G A A G A A C G C A G 3'	Species per cluster	Cluster number
AY308582	Hebeloma	aestivale			460	1
AY311522	Hebeloma	aff			460	1
AY308583	Hebeloma	albocolossum			460	1
AY308584	Hebeloma	alpinum			460	1
AY308585	Hebeloma	ammophilum			460	1
AY308586	Hebeloma	atrobrunneum			460	1
AY309958	Hebeloma	bruchetii			460	1
AY309959	Hebeloma	brunneifolium			460	1
AY309960	Hebeloma	bryogenes			460	1
AF124670	Hebeloma	cavipes			460	1
AF124699	Hebeloma	circinans			460	1
AF124691	Hebeloma	collariatum			460	1
AF124716	Hebeloma	crustuliniforme			460	1
AF325643	Hebeloma	fastibile			460	1
AY309964	Hebeloma	fragilipes			460	1
AY311516	Hebeloma	helodes			460	1
AF124669	Hebeloma	hiemale			460	1
AF430291	Hebeloma	incarnatulum			460	1
AY311517	Hebeloma	leucosarx			460	1
AY311518	Hebeloma	longicaudum			460	1
AY311519	Hebeloma	malenconii			460	1
AY311520	Hebeloma	marginatulum			460	1
AY311521	Hebeloma	mesophaeum			460	1
AY311523	Hebeloma	monticola			460	1
AY311524	Hebeloma	nigellum			460	1
AY311525	Hebeloma	oculatum			460	1
AY312976	Hebeloma	pallidum			460	1
AY312977	Hebeloma	polare			460	1
AY312979	Hebeloma	populinum			460	1
AY312980	Hebeloma	psammophilum			460	1
AF124706	Hebeloma	pusillum			460	1
AY278767	Hebeloma	radicosum			460	1
AY312983	Hebeloma	remyi			460	1
AY312984	Hebeloma	repandum			460	1
AY312985	Hebeloma	sacchariolens			460	1
AY312986	Hebeloma	saliciphilum			460	1
AF124715	Hebeloma	sarcophyllum			460	1
AF124682	Hebeloma	sinapizans			460	1
AY320390	Hebeloma	sp			460	1
AY320393	Hebeloma	subconcolor			460	1
AY320394	Hebeloma	subsaponaceum			460	1
AY320395	Hebeloma	testaceum			460	1
AF124701	Hebeloma	truncatum			460	1
AY320396	Hebeloma	vaccinum			460	1
AF430250	Hebeloma	velutipes			460	1
AY320397	Hebeloma	versipelle			460	1
AY320398	Hebeloma	vinosophyllum			460	1
AY188367	Hormonema	aff			460	1
AY616210	Hormonema	carpetanum			460	1
AF462439	Hormonema	dematioides			460	1
HMA244247	Hormonema	macrosporum			460	1
AY616213	Hormonema	prunorum			460	1
AY465454	Hormonema	sp			460	1
AF325602	Hymenogaster	remyi			460	1
AB027385	Hypomyces	chrysospermus			460	1
HMA494836	Hypsizygus	marmoreus			460	1
AF335452	Inocybe	cf			460	1
AY251309	Inonotus	hispida			460	1
AY616212	Kabatina	juniperi			460	1
AF013226	Kabatina	thujae			460	1
AY198400	Kluyveromyces	delphensis			460	1
AY325966	Kluyveromyces	hubeiensis			460	1
AF444425	Kockovaella	imperatae			460	1
AB052560	Kockovaella	sacchari			460	1

Accession Number	Genus	5.8S Species	Primer	Sequence	Species per cluster	Cluster number
			58A2R	3' C G A T G A A G A A C G C		
			58A1F	A T G A A G A A C G C A G 3'		
AB052559	Kockovaella	thailandica			460	1
AF345807	Kuehneromyces	mutabilis			460	1
AF539737	Laccaria	amethystea			460	1
AF440665	Laccaria	amethystina			460	1
LBAF6598	Laccaria	bicolor			460	1
AY228356	Laccaria	cf			460	1
LLAF6596	Laccaria	laccata			460	1
AY750156	Laccaria	proxima			460	1
LAC534899	Laccaria	sp			460	1
LAC278139	Lactarius	acerrimus			460	1
AY606944	Lactarius	blennius			460	1
AY606945	Lactarius	camphoratus			460	1
AF096983	Lactarius	chrysorrheus			460	1
LCO272246	Lactarius	controversus			460	1
AF249284	Lactarius	deliciosus			460	1
AF249286	Lactarius	deterrimus			460	1
AY750160	Lactarius	fallax			460	1
AY606946	Lactarius	helvus			460	1
LIN272243	Lactarius	insulsus			460	1
AF140256	Lactarius	intermedius			460	1
AY606948	Lactarius	lilacinus			460	1
AY336952	Lactarius	mairei			460	1
AY606950	Lactarius	necator			460	1
AY606951	Lactarius	pallidus			460	1
AY534201	Lactarius	pseudomucidus			460	1
AY331013	Lactarius	pterosporus			460	1
AY606953	Lactarius	pubescens			460	1
AF140269	Lactarius	quieticolor			460	1
LQU272247	Lactarius	quietus			460	1
AY331011	Lactarius	repraesentaneus			460	1
AY292987	Lactarius	rubrozonatus			460	1
AY606954	Lactarius	ruginosus			460	1
AF249288	Lactarius	salmonicolor			460	1
AF249291	Lactarius	sanguifluus			460	1
AY336957	Lactarius	scoticus			460	1
AF140263	Lactarius	scrobiculatus			460	1
AF249292	Lactarius	semisanguifluus			460	1
AF448488	Lactarius	sp			460	1
AY336961	Lactarius	spinosulus			460	1
AF140261	Lactarius	subsericatus			460	1
AY336954	Lactarius	torminosulus			460	1
AY336959	Lactarius	torminosus			460	1
LTR534935	Lactarius	trivialis			460	1
LUT534936	Lactarius	uvidus			460	1
AY332549	Lactarius	vinosus			460	1
AF096979	Lactarius	zonarius			460	1
AF502835	Leaf	litter			460	1
LMO419917	Leucogyrophana	pinastri			460	1
AF357052	Lyophyllum	caerulescens			460	1
AF357060	Lyophyllum	decastes			460	1
AF357051	Lyophyllum	sykosporum			460	1
AF482853	Macrolepiota	velosa			460	1
AB026819	Magnaporthe	grisea			460	1
AY256708	Marasmiellus	juniperinus			460	1
AY559363	Melanized	limestone			460	1
MAM555514	Melanogaster	ambiguus			460	1
MBR555532	Melanogaster	broomeianus			460	1
MCF555521	Melanogaster	cf			460	1
MIN555515	Melanogaster	intermedius			460	1
MMA555528	Melanogaster	macrosporus			460	1
MTU555527	Melanogaster	tuberiformis			460	1
MVA419204	Melanogaster	variegatus			460	1
MVI555525	Melanogaster	vittadinii			460	1
AF138269	Metarhizium	flavoviride			460	1

Accession Number	Genus	5.8S Species	58A2R / 58A1F	3' C G A T G A A G A A C G C / A T G A A G A A C G C A G 3'	Species per cluster	Cluster number
AF486120	Mycelium	radicis			460	1
AF178415	Nectria	borneensis			460	1
AF059207	Nectria	haematococca			460	1
AF178398	Nectria	ipomoeae			460	1
AF178412	Neocosmospora	africana			460	1
AF178413	Neocosmospora	ornamentata			460	1
AY381143	Neocosmospora	vasinfecta			460	1
AY373895	Neosartorya	fischeri			460	1
AF459730	Neosartorya	quadricincta			460	1
AF459733	Neosartorya	stramenia			460	1
U66443	Omphalina	grisella			460	1
U66450	Omphalina	pyxidata			460	1
U66451	Omphalina	rivulicola			460	1
POC504645	Paraglomus	sp			460	1
AF167688	Paxillus	filamentosus			460	1
AY585921	Paxillus	involutus			460	1
AF167689	Paxillus	vernalis			460	1
AF033401	Penicillium	adametzii			460	1
AF033412	Penicillium	asperosporum			460	1
AF033402	Penicillium	bilaiae			460	1
AF481122	Penicillium	boreae			460	1
AF484399	Penicillium	brocae			460	1
AF033427	Penicillium	cyaneum			460	1
AF033445	Penicillium	donkii			460	1
AF033443	Penicillium	fuscum			460	1
AF125941	Penicillium	geastrivorus			460	1
AY373915	Penicillium	glabrum			460	1
AF454077	Penicillium	isariiforme			460	1
AY373921	Penicillium	janthinellum			460	1
AY373922	Penicillium	lividum			460	1
AF527058	Penicillium	montanense			460	1
AF033438	Penicillium	oxalicum			460	1
AF033408	Penicillium	purpurescens			460	1
AF033433	Penicillium	raperi			460	1
AF033439	Penicillium	rolfsii			460	1
AY373930	Penicillium	sclerotiorum			460	1
AF481123	Penicillium	sp			460	1
AY373933	Penicillium	spinulosum			460	1
AY373934	Penicillium	thomii			460	1
AF033461	Penicillium	vinaceum			460	1
AY465505	Pezizales	sp			460	1
U66436	Phaeotellus	griseopallidus			460	1
AY664502	Phialocephala	fortinii			460	1
AY618244	Phialocephala	sp			460	1
AF374717	Pisolithus	tinctorius			460	1
AF516562	Polyporus	dictyopus			460	1
AF516568	Polyporus	melanopus			460	1
AF511442	Polyporus	tubiformis			460	1
AF423121	Postia	placenta			460	1
PSM244257	Pringsheimia	smilacis			460	1
AF325561	Protoglossum	sp			460	1
AB158635	Psilocybe	fasciata			460	1
AY219389	Rhizochaete	brunnea			460	1
AF377154	Rhizopogon	alkalivirens			460	1
AF366387	Rhizopogon	diabolicus			460	1
AF224276	Rhizopogon	ellenae			460	1
AF377123	Rhizopogon	idahoensis			460	1
AF377134	Rhizopogon	kauffmanii			460	1
AF377126	Rhizopogon	pedicellus			460	1
AF377137	Rhizopogon	pinyonensis			460	1
AF058315	Rhizopogon	roseolus			460	1
RRU277645	Rhizopogon	rubescens			460	1
AF377157	Rhizopogon	salebrosus			460	1
AF377119	Rhizopogon	semireticulatus			460	1

Accession Number	Genus	Species (5.8S)	Primer	Sequence	Species per cluster	Cluster number
			58A2R	3' C G A T G A A G A A C G C		
			58A1F	A T G A A G A A C G C A G 3'		
AF377108	Rhizopogon	sp			460	1
AF377152	Rhizopogon	subbadius			460	1
RSRRITSA	Rhizopogon	subcaerulescens			460	1
AF377120	Rhizopogon	subcaerulescens			460	1
AF377133	Rhizopogon	subpurpurascens			460	1
AF366392	Rhizopogon	vesiculosus			460	1
AF263931	Rhizopogon	vinicolor			460	1
AF062934	Rhizopogon	vulgaris			460	1
AY183366	Rhizosphaera	kalkhoffii			460	1
AF462432	Rhizosphaera	kobayashii			460	1
AF462431	Rhizosphaera	macrospora			460	1
AF462430	Rhizosphaera	oudemansii			460	1
AY183365	Rhizosphaera	pini			460	1
AF462433	Rhizosphaera	sp			460	1
SPRRNA	Saccharomyces	cerevisiae			460	1
AF485074	Sarcosomataceous	endophyte			460	1
SJRIBRNA	Schizosaccharomyces	japonicus			460	1
SP18RRNAA	Schizosaccharomyces	pombe			460	1
AF462438	Sclerophoma	pityophila			460	1
AY220838	Scutellinia	colensoi			460	1
AY293059	Selenophoma	eucalypti			460	1
AF054866	Sepedonium	chlorinum			460	1
SHI536024	Serpula	himantioides			460	1
SLA557318	Serpula	lacrymans			460	1
AY293060	Sphaerulina	eucalypti			460	1
AY332486	Stenocarpella	maydis			460	1
AF485076	Strumella	coryneoidea			460	1
AY781224	Sydowia	polyspora			460	1
AF357057	Tephrocybe	ambusta			460	1
AY260098	Teratosphaeria	microspora			460	1
AF321366	Termitomyces	eurhizus			460	1
AB051880	Termitomyces	sp			460	1
AF321367	Termitomyces	striatus			460	1
AF325577	Thaxterogaster	porphyreum			460	1
TSU83471	Thelephoraceae	sp			460	1
AF430259	Thelephoraceous	ectomycorrh			460	1
AF272904	Tomentella	atramentaria			460	1
AF272937	Tomentella	badia			460	1
AF272912	Tomentella	botryoides			460	1
AF272908	Tomentella	bryophila			460	1
AY010274	Tomentella	cf			460	1
AF272909	Tomentella	ferruginea			460	1
AF272942	Tomentella	fusco-cinerea			460	1
AF272941	Tomentella	lapidum			460	1
AF272910	Tomentella	lilacinogrisea			460	1
AF272925	Tomentella	pilosa			460	1
AF272943	Tomentella	punicea			460	1
TRU83480	Tomentella	ramosissima			460	1
TOM534917	Tomentella	sp			460	1
AY635177	Tomentella	stuposa			460	1
AY010275	Tomentella	subclavigera			460	1
AF272920	Tomentella	umbrinospora			460	1
AB100609	Torrubiella	flava			460	1
AY309019	Trametes	versicolor			460	1
U63475	Trichaptum	abietinum			460	1
U63476	Trichaptum	biforme			460	1
U63478	Trichaptum	fusco-violaceum			460	1
U63477	Trichaptum	laricinum			460	1
AF377185	Tricholoma	griseoviolaceum			460	1
TMU62964	Tricholoma	matsutake			460	1
AF349686	Tricholoma	portentosum			460	1
AY573536	Tricholoma	scalpturatum			460	1
AF377184	Tricholoma	sp			460	1
AF241514	Tricholoma	unifactum			460	1

Accession	5.8S		58A2R	3'	C	G	A	T	G	A	A	G	A	A	C	G	C				Species	Cluster
Number	Genus	Species	58A1F				A	T	G	A	A	G	A	A	C	G	C	A	G	3'	per cluster	number
AB164370	Trichosporon	siamense																			460	1
AF414693	Trichosporon	veenhuisii																			460	1
TAB244267	Trimmatostroma	abietina																			460	1
AY559362	Trimmatostroma	abietis																			460	1
TSA244264	Trimmatostroma	salicis																			460	1
AY329595	Trogia	sp																			460	1
TME459582	Tuber	melanosporum																			460	1
AY456371	Tylopilus	sp																			460	1
AF485077	Urnula	helvelloides																			460	1
XAR419221	Xerocomus	armeniacus																			460	1
XCH419223	Xerocomus	chrysenteron																			460	1
AF402140	Xerocomus	pruinatus																			460	1
CGE510274	Cenococcum	geophilum													-						22	2
AF297229	Cercospora	asparagi													-						22	2
AF297222	Cercospora	beticola													-						22	2
AF297226	Cercospora	kalmiae													-						22	2
AY633838	Cercospora	kikuchii													-						22	2
AF297230	Cercospora	nicotianae													-						22	2
AF297232	Cercospora	sorghi													-						22	2
GLA301970	Glomerella	lagenaria													-						22	2
AF297236	Mycosphaerella	brassicicola													-						22	2
AY266153	Mycosphaerella	cruenta													-						22	2
AF181705	Mycosphaerella	fijiensis													-						22	2
AF309603	Mycosphaerella	flexuosa													-						22	2
AF468881	Mycosphaerella	juvenis													-						22	2
AY152570	Mycosphaerella	linicola													-						22	2
AY725575	Mycosphaerella	ohnowa													-						22	2
AF079776	Phomopsis	amaranthicola													-						22	2
AF309596	Pseudocercospora	paraguayen													-						22	2
AY259133	Ramulispora	sorghi													-						22	2
AY152571	Septoria	aciculosa													-						22	2
AY152569	Septoria	gerberae													-						22	2
AY826767	Septoria	glycines													-						22	2
AY260082	Septoria	protearum													-						22	2
AY318746	Pisolithus	albus											G								2	3
AF270781	Pisolithus	sp											G								2	3
CFR486964	Calvatia	fragilis				C															1	4
AF058303	Rhizopogon	burlinghamii							A												1	5
AF096976	Pisolithus	arhizus										A						N			1	6
AF333489	Phakopsora	pachyrhizi														A					1	7
AF097575	Elsinoe	leucospermi												-							1	8
AF241480	Acer	argutum															T				75	9
AF241481	Acer	buergerianum															T				75	9
AF406969	Acer	caesium															T				75	9
AF401158	Acer	campestre															T				75	9
AF401138	Acer	cappadocicum															T				75	9
AF241482	Acer	carpinifolium															T				75	9
AF241483	Acer	cissifolium															T				75	9
AF401135	Acer	crassum															T				75	9
AF020368	Acer	crataegifolium															T				75	9
AF401144	Acer	davidii															T				75	9
AF241484	Acer	diabolicum															T				75	9
AF020379	Acer	distylum															T				75	9
AF241486	Acer	fabri															T				75	9
AF241488	Acer	glabrum															T				75	9
AF401141	Acer	henryi															T				75	9
AF241489	Acer	japonicum															T				75	9
AF401143	Acer	kungshanense															T				75	9
AF241490	Acer	laurinum															T				75	9
AF401156	Acer	macrophyllum															T				75	9
AF401129	Acer	mandshuricum															T				75	9
AF401124	Acer	miaoshanicum															T				75	9
AF020369	Acer	micranthum															T				75	9
AF241491	Acer	mono															T				75	9

Accession Number	Genus	5.8S Species	58A2R: 3' C G A T G A A G A A C G C / 58A1F: A T G A A G A A C G C A G 3'	Species per cluster	Cluster number
AF401127	Acer	monspessulanum	T	75	9
AF241492	Acer	negundo	T	75	9
AF020380	Acer	nipponicum	T	75	9
AF241494	Acer	oblongum	T	75	9
AF241495	Acer	okamotoanum	T	75	9
AF401128	Acer	opalus	T	75	9
AF241496	Acer	palmatum	T	75	9
AF401132	Acer	paxii	T	75	9
AF241497	Acer	pensylvanicum	T	75	9
AF241498	Acer	pentaphyllum	T	75	9
AF241499	Acer	platanoides	T	75	9
AF401134	Acer	poliophyllum	T	75	9
AF241500	Acer	pseudoplatanus	T	75	9
AF241501	Acer	pseudosieboldianum	T	75	9
AF401125	Acer	pubinerve	T	75	9
AF241502	Acer	rubrum	T	75	9
AF020371	Acer	rufinerve	T	75	9
AF401151	Acer	saccharinum	T	75	9
AF401152	Acer	saccharum	T	75	9
AF020376	Acer	shirasawanum	T	75	9
AF020377	Acer	sieboldianum	T	75	9
AF241503	Acer	spicatum	T	75	9
AF241504	Acer	takesimense	T	75	9
AF241487	Acer	tataricum	T	75	9
AF241505	Acer	tegmentosum	T	75	9
AF401154	Acer	tetramerum	T	75	9
AF401126	Acer	trautvetteri	T	75	9
AF241506	Acer	triflorum	T	75	9
AF241507	Acer	truncatum	T	75	9
AF020372	Acer	tschonoskii	T	75	9
AF401159	Acer	wardii	T	75	9
AF080029	Bursera	tecomaca	T	75	9
AF401120	Dipteronia	dyeriana	T	75	9
AF241508	Dipteronia	sinensis	T	75	9
AY523434	Larix	decidua	T	75	9
AF538064	Larix	gmelinii	T	75	9
AY523413	Larix	griffithiana	T	75	9
AF538068	Larix	himalaica	T	75	9
AY523445	Larix	kaempferi	T	75	9
AF041348	Larix	laricina	T	75	9
AF041346	Larix	lyallii	T	75	9
AF538067	Larix	mastersiana	T	75	9
AY523455	Larix	occidentalis	T	75	9
AF538060	Larix	potaninii	T	75	9
AY523450	Larix	sibirica	T	75	9
AF538063	Larix	speciosa	T	75	9
AY641510	Malosma	laurina	T	75	9
AF041352	Pseudotsuga	japonica	T	75	9
AF041354	Pseudotsuga	macrocarpa	T	75	9
AF041353	Pseudotsuga	menziesii	T	75	9
AF041350	Pseudotsuga	sinensis	T	75	9
AF041351	Pseudotsuga	wilsoniana	T	75	9
AF289045	Apedinella	radians		68	10
BFO491208	Begonia	formosana		68	10
BRA491250	Begonia	ravenii		68	10
BSU581914	Botryococcus	sp		68	10
AY206424	Caulerpa	cupressoides		68	10
AY206426	Caulerpa	webbiana		68	10
CE58S25S	Chlorella	ellipsoidea		68	10
AF479758	Chlorella	sp		68	10
AB162910	Chlorella	vulgaris		68	10
AF156602	Chloromonas	playfairii		68	10
AY476827	Chlorothrix	sp		68	10
AY198124	Collinsiella	tuberculata		68	10

5.8S

Accession Number	Genus	Species																		Species per cluster	Cluster number
			58A2R 3'	C	G	A	T	G	A	A	G	A	A	C	G	C					
			58A1F			A	T	G	A	A	G	A	A	C	G	C	A	G	3'		
AF087105	Compsopogon	coeruleus																		68	10
AF154863	Cosmarium	fontrabiense																		68	10
AF154856	Cosmarium	obtusatum																		68	10
AF154862	Cosmarium	tumidum																		68	10
AY548208	Cynanchum	auriculatum																		68	10
AF033280	Dysmorphococcus	globosus																		68	10
U71248	Elaeis	oleifera																		68	10
EIAJ211	Enteromorpha	intestinalis																		68	10
FHRRNA	Funaria	hygrometrica																		68	10
GPRRNAS	G	planctonica																		68	10
AY497560	Ganonema	farinosum																		68	10
AY497564	Helminthocladia	australis																		68	10
AY083659	Heritiera	littoralis																		68	10
AY328172	Hopea	hainanensis																		68	10
AF082342	Laurencia	perforata																		68	10
AF082340	Laurencia	sp																		68	10
AF082345	Laurencia	viridis																		68	10
AY497565	Liagora	boergesenii																		68	10
AY497567	Liagora	ceranoides																		68	10
AY497568	Liagora	decussata																		68	10
AY497570	Liagora	orientalis																		68	10
AY497572	Liagora	segawae																		68	10
AY497576	Liagora	valida																		68	10
AF455748	Lycium	chinense																		68	10
AY880681	Lycium	ruthenicum																		68	10
MGRDNA	M	glaucescens																		68	10
AF448792	Marsilea	quadrifolia																		68	10
AF415173	Monostroma	angicava																		68	10
AF415171	Monostroma	arcticum																		68	10
AF428049	Monostroma	grevillei																		68	10
AF163111	Monostroma	nitidum																		68	10
PBRRNAS	P	basiliense																		68	10
PPRRNAS	P	paucicellulare																		68	10
PSRRNAS	P	sarcinoidea																		68	10
AB162911	Parachlorella	kessleri																		68	10
AY548209	Pleuropterus	multiflorus																		68	10
AF033296	Pyrobotrys	stellata																		68	10
AF452163	Pythium	ultimum																		68	10
SSRRAA	S	similis																		68	10
AF448791	Salvinia	natans																		68	10
SAC249511	Scenedesmus	acuminatus																		68	10
SAC249508	Scenedesmus	acutus																		68	10
AY510467	Scenedesmus	bajacalifornicus																		68	10
AY510470	Scenedesmus	deserticola																		68	10
AY510466	Scenedesmus	dissociatus																		68	10
SNA249510	Scenedesmus	naegelii																		68	10
SOB249505	Scenedesmus	obliquus																		68	10
AY510465	Scenedesmus	rotundus																		68	10
AF154855	Staurastrum	sp																		68	10
UZRRNAS	U	zonata																		68	10
AJ626846	Uncultured	Urospora																		68	10
AY476821	Urospora	neglecta																		68	10
AY476812	Urospora	sp																		68	10
AY476814	Urospora	wormskioldii																		68	10
AY328171	Vatica	mangachapoi																		68	10
AY497577	Yamadaella	caenomyce																		68	10
AF401149	Acer	decandrum														T		C		1	11

Accession	28S		3' end										NLB4										3' end											NLC2											Species	Cluster
Number	Genus	Species	G	T	C	A	T	A	G	A	G	G	G	T	G	A	G	A	A	T	C	C	G	A	G	T	T	G	T	T	T	G	G	G	A	A	T	G	C	A	G	C	T	C	per cluster	number
AF433107	Aspergillus	elegans																																									494	1
AF335451	Boletus	mirabilis																																									494	1
AY394919	Cenococcum	geophilum																																									494	1
AF325292	Lactarius	torminosus																																									494	1
SCRRM01	Saccharomyces	cerevisiae																																									494	1
AF130379	Fusarium	dimerum		C															G	C							A													T					76	2
AF049176	Verticillium	lecanii		C															G	C							A													T					76	2
AY612800	Boletus	bicolor				G																																					49	3
AF071536	Suillus	sinuspaulianus				G																																					49	3
AF275540	Microascus	giganteus		C			C												G	C							A													T					19	4
AY216676	Aspergillus	ustus					G																																				20	5
AF042571	Russula	earlei	A																																								11	7
AF042622	Suillus	luteus				G											C																										7	8
AF506462	Russula	nauseosa					C																																				7	9
AF291349	Myxarium	grilletii	T																																								7	10
AB027371	Cordyceps	kanzashiana		C			C												G	C				G			A													T			C		4	11
AF218549	Russula	silvicola							N																																		4	12
AY612804	Boletus	subvelutipes				G	C																																				4	13
AF274100	Pertusaria	erythrella				C																																					3	15
AF479200	Abelia	triflora		C		G	G												G	C				G																			C		93	62
AF297535	Cornus	mas		C		G	G												G	C				G																			C		93	62
AF479183	Helianthus	annuus		C		G	G												G	C				G																			C		93	62
AY056503	Thuja	plicata		C		G	G												G	C				G																			C		93	62
AF389239	Aextoxicon	punctatum		C			G												G	C				G																			C		43	63
SA25SR	Sinapis	alba		C			G												G	C				G																			C		43	63
AY056502	Larix	leptolepis		C		T	G										C		G	C				G																			C		9	64
AY189036	Aralidium	pinnatifidum		C		T	G												G	C				G																			C		7	65
AAU90690	Araucaria	araucana		C		G	G												G	C				G																	A		C		6	66
AGU90683	Abies	grandis		C		T	G										T		G	C				G																			C		4	67

Accession Number	28S Genus	Species	3' end NLB4 G T C A T A G A G G G T G A G A A T C C	3' end NLC2 G A G T T G T T T G G G A A T G C A G C T C	Species per cluster	Cluster number
ABI406527	Abortiporus	biennis			494	1
AY039305	Acanthobasidium	phragmitis			494	1
AY039333	Acanthofungus	rimosus			494	1
AF506400	Acanthophysellum	lividocoer			494	1
AY039327	Acanthophysium	bisporum			494	1
AY039310	Acanthophysium	cerussatum			494	1
AY039319	Acanthophysium	lividocaerul			494	1
AY039322	Acanthophysium	weirii			494	1
AY584643	Acarosporina	microspora			494	1
AF261623	Agaricales	sp			494	1
AF367935	Agrocybe	firma			494	1
AY646101	Agrocybe	praecox			494	1
AY039331	Aleurobotrys	botryosus			494	1
AF506403	Aleurocystidiellum	subcruen			494	1
AY039324	Aleurodiscus	abietis			494	1
AF506399	Aleurodiscus	cerussatus			494	1
AY039320	Aleurodiscus	lapponicus			494	1
AY039321	Aleurodiscus	laurentianus			494	1
AY039332	Aleurodiscus	mirabilis			494	1
AY586625	Amaurodon	viridis			494	1
AF518597	Amphinema	byssoides			494	1
AY586628	Amylocorticium	subincarnatu			494	1
AF518598	Amylocystis	lapponica			494	1
AF506405	Amylostereum	areolatum			494	1
AF518599	Amylostereum	chailletii			494	1
AF506407	Amylostereum	laevigatum			494	1
AF287844	Antrodia	carbonica			494	1
AJ583430	Antrodia	xantha			494	1
AF433074	Aspergillus	auricomus			494	1
AA15508	Aspergillus	avenaceus			494	1
AF433046	Aspergillus	bridgeri			494	1
AB29823	Aspergillus	brunneo-uniseriatu			494	1
AD15491	Aspergillus	dimorphicus			494	1
AF433107	Aspergillus	elegans			494	1
AF15504	Aspergillus	flaschentraegeri			494	1
AG15490	Aspergillus	gorakhpurensis			494	1
AI15485	Aspergillus	itaconicus			494	1
AF433120	Aspergillus	melleus			494	1
AF433088	Aspergillus	ochraceus			494	1
AF433084	Aspergillus	ostianus			494	1
AF433104	Aspergillus	petrakii			494	1
AP20822	Aspergillus	pulvinus			494	1
AY338959	Aspergillus	sclerotiorum			494	1
AF433121	Aspergillus	sulphureus			494	1
AW15495	Aspergillus	wentii			494	1
AY586631	Asterodon	ferruginosum			494	1
AY293168	Athelia	epiphylla			494	1
AY700189	Aureoboletus	thibetanus			494	1
AF291289	Auricularia	auricula-judae			494	1
AF291290	Auricularia	delicata			494	1
AF291291	Auricularia	fuscosuccinea			494	1
AF506492	Auricularia	mesenterica			494	1
AF261554	Auricularia	polytricha			494	1
AY634277	Auricularia	sp			494	1
AY612797	Austroboletus	betula			494	1
AY612798	Austroboletus	mucosus			494	1
AF291294	Basidiodendron	caesiocinere			494	1
AF291297	Basidiodendron	grandinioide			494	1
AY187277	Basidiomycete	sp			494	1
AB096738	Bjerkandera	adusta			494	1
AY089741	Bjerkandera	adustus			494	1
AY633927	Bjerkandera	sp			494	1
AF506376	Boidinia	furfuracea			494	1
AF506379	Boidinia	propinqua			494	1
AF506378	Boidinia	sp			494	1
AF050642	Boletellus	betula			494	1
AF050652	Boletellus	mirabilis			494	1
AY684158	Boletellus	projectellus			494	1
AF139684	Boletus	eximius			494	1
AF456834	Boletus	griseus			494	1
AF335451	Boletus	mirabilis			494	1
AF456811	Boletus	retipes			494	1
AF287849	Bondarzewia	berkeleyi			494	1
AF393048	Botryobasidium	subcoronatum			494	1
AY004336	Botryosphaeria	ribis			494	1
AY586640	Byssomerulius	corium			494	1
BSP406534	Byssomerulius	sp			494	1
AF279885	Caloplaca	cerina			494	1
AY394919	Cenococcum	geophilum			494	1

Accession Number	28S Genus	Species	3' end NLB4 GTCATAGAGGGTGAGAATCC	3' end NLC2 GAGTTGTTTGGGAATGCAGCTC	Species per cluster	Cluster number
AF518606	Ceraceomyces	serpens			494	1
AF393049	Ceriporia	viridans			494	1
CC15507	Chaetosartorya	chrysella			494	1
CC15505	Chaetosartorya	cremea			494	1
CS15512	Chaetosartorya	stromatoides			494	1
AB075359	Chromocleista	cinnabarina			494	1
AY684165	Climacodon	septentrionalis			494	1
AY647208	Clitocybe	subvelosa			494	1
AF506382	Conferticium	ravum			494	1
AY586649	Coniophora	arida			494	1
AJ583426	Coniophora	puteana			494	1
CMI406565	Coprinus	micaceus			494	1
AY219581	Cortinarius	abnormis			494	1
AY033127	Cortinarius	alboroseus			494	1
AY174807	Cortinarius	anserinus			494	1
AF539730	Cortinarius	austroturmalis			494	1
AF388786	Cortinarius	badiovinaceus			494	1
AY174861	Cortinarius	boudieri			494	1
AF539715	Cortinarius	caelicolor			494	1
AF539711	Cortinarius	cervinus			494	1
AF388761	Cortinarius	cinereobrunneus			494	1
AY174812	Cortinarius	cumatilis			494	1
AF539725	Cortinarius	elaphinus			494	1
AF539716	Cortinarius	flammuloides			494	1
AY174787	Cortinarius	glaucopus			494	1
AF388775	Cortinarius	globuliformis			494	1
AF539720	Cortinarius	icterinus			494	1
AY174846	Cortinarius	multiformis			494	1
AF539733	Cortinarius	myxoclaricolor			494	1
AF539727	Cortinarius	pachynemeus			494	1
AY174854	Cortinarius	porphyropus			494	1
AY174858	Cortinarius	purpurascens			494	1
AY174800	Cortinarius	saginus			494	1
AF539729	Cortinarius	squamiger			494	1
AF539717	Cortinarius	viridibasalis			494	1
AY174789	Cortinarius	viridocoeruleus			494	1
AF393050	Cryptoporus	volvatus			494	1
AY293616	Cyttarophyllum	besseyi			494	1
AY293176	Dacryobolus	sudans			494	1
AF291313	Daedalea	quercina			494	1
DMO406548	Datronia	mollis			494	1
DSQ487514	Dichomitus	squalens			494	1
AF506428	Dichostereum	aff			494	1
AF506391	Dichostereum	granulosum			494	1
AF506392	Dichostereum	pallescens			494	1
AJ583428	Donkioporia	expansa			494	1
AF506430	Echinodontium	tinctorium			494	1
AF476997	Ectomycorrhizal	root			494	1
AF291318	Eichleriella	deglubens			494	1
AY586653	Elmerina	holophaea			494	1
AY505543	Endoperplexa	enodulosa			494	1
AF033435	Eupenicillium	brefeldianum			494	1
AY213698	Eupenicillium	cinnamopurpur			494	1
AF033462	Eupenicillium	rubidurum			494	1
AY243524	Exidia	crenata			494	1
AF506493	Exidia	glandulosa			494	1
AF291320	Exidia	japonica			494	1
AF291321	Exidia	pithya			494	1
AF347112	Exidia	recisa			494	1
AF291323	Exidia	saccharina			494	1
AY700191	Exidia	sp			494	1
AF291325	Exidia	truncata			494	1
AY645056	Exidia	uvapsassa			494	1
AY586654	Exidiopsis	calcea			494	1
AF291328	Exidiopsis	grisea			494	1
AF395309	Exidiopsis	plumbescens			494	1
FRU406475	Fibricium	rude			494	1
AF195588	Flammula	alnicola			494	1
AY380408	Flammulaster	sp			494	1
AF291331	Fomes	fomentarius			494	1
AF311048	Fomitopsis	pinicola			494	1
AY618209	Fuscoporia	palmicola			494	1
GBRS25S	G	boninense			494	1
GLRZ25S	G	lucidum			494	1
GM25S9	G	microsporum			494	1
GTJ225S	G	tsugae			494	1
AF195590	Galerina	marginata			494	1
AF261653	Galerina	paludosa			494	1
AF287859	Geastrum	saccatum			494	1

Accession Number	28S Genus	Species	3' end NLB4 GTCATAGAGGGTGAGAATCC	3' end NLC2 GAGTTGTTTGGGAATGCAGCTC	Species per cluster	Cluster number
AF141612	Gelatoporia	pannocincta			494	1
AF033389	Geosmithia	argillacea			494	1
AF033386	Geosmithia	cylindrospora			494	1
AF033387	Geosmithia	emersonii			494	1
AB047228	Geosmithia	viridis			494	1
U66434	Gerronema	subclavatum			494	1
AF506433	Gloeocystidiellum	aculeatum			494	1
AF506432	Gloeocystidiellum	aspellum			494	1
AF506434	Gloeocystidiellum	compactum			494	1
AF506439	Gloeocystidiellum	formosanu			494	1
AF506381	Gloeocystidiellum	heimii			494	1
AF287860	Gloeocystidiellum	leucoxant			494	1
AF506421	Gloeocystidiellum	luridum			494	1
AF310090	Gloeocystidiellum	sp			494	1
AF506404	Gloeocystidiellum	subasperi			494	1
AF506442	Gloeocystidiellum	triste			494	1
AF506443	Gloeocystidiellum	wakullum			494	1
AF506438	Gloeocystidiopsis	aff			494	1
AF506437	Gloeocystidiopsis	flammea			494	1
AF506445	Gloeodontia	discolor			494	1
AF506448	Gloeomyces	graminicola			494	1
AF506436	Gloeopeniophorella	aff			494	1
AF506435	Gloeopeniophorella	convolve			494	1
AF506440	Gloeopeniophorella	laxa			494	1
AY586656	Gloeoporus	taxicola			494	1
AF506450	Gloiodon	nigrescens			494	1
AY640951	Glypholecia	scabra			494	1
AY647207	Gomphus	clavatus			494	1
GFU406506	Grammothele	fuligo			494	1
AF287863	Grifola	frondosa			494	1
AF265538	Gymnomyces	pallidus			494	1
HC11918	Hebeloma	crustuliniforme			494	1
AY033139	Hebeloma	fastibile			494	1
AF430291	Hebeloma	incarnatulum			494	1
AF261515	Hebeloma	longicaudum			494	1
AY038310	Hebeloma	olympianum			494	1
AY219607	Hebeloma	sp			494	1
AY745703	Hebeloma	velutipes			494	1
AF261647	Hemipholiota	destruens			494	1
AF261646	Hemipholiota	lucifera			494	1
AF195593	Hemipholiota	populnea			494	1
AF347096	Heterobasidion	annosum			494	1
AF291334	Heterochaete	hirneoloides			494	1
AF291335	Heterochaete	shearii			494	1
AF291336	Heterochaete	sp			494	1
AY509552	Heterochaetella	brachyspora			494	1
HHY487625	Hexagonia	hydnoides			494	1
AF139956	Hohenbuehelia	petalodes			494	1
AF042601	Hohenbuehelia	sp			494	1
AF135171	Hohenbuehelia	tristis			494	1
AY684156	Hygrophoropsis	aurantiaca			494	1
HJE406452	Hyphodontia	barbajovis			494	1
AY646100	Hyphodontia	gossypina			494	1
AF195595	Hypholoma	capnoides			494	1
AF261628	Hypholoma	ericaeum			494	1
AF261630	Hypholoma	fasciculare			494	1
AY635774	Hypholoma	sublateritium			494	1
AF261631	Hypholoma	subviride			494	1
AF261627	Hypholoma	udum			494	1
HEI406508	Hypochnicium	eichleri			494	1
AY293189	Hypsizygus	tessulatus			494	1
AY059032	Inonotus	glomeratus			494	1
AF291341	Inonotus	nodulosus			494	1
AF311017	Inonotus	obliquus			494	1
AY059040	Inonotus	weirii			494	1
IBE406543	Ischnoderma	benzoinum			494	1
IRE487927	Ischnoderma	resinosum			494	1
AF518625	Junghuhnia	subundata			494	1
AY582127	Kazachstania	aerobia			494	1
AF398482	Kazachstania	viticola			494	1
AY048159	Kluyveromyces	africanus			494	1
AY048170	Kluyveromyces	yarrowii			494	1
AF291342	Kuehneromyces	mutabilis			494	1
AF325268	Lactarius	badiosanguineus			494	1
AF325269	Lactarius	blennius			494	1
AF325270	Lactarius	camphoratus			494	1
AF325271	Lactarius	circellatus			494	1
LC11919	Lactarius	corrugis			494	1
AY631899	Lactarius	deceptivus			494	1

Accession Number	28S Genus	Species	3' end NLB4 GTCATAGAGGGTGAGAATCC	3' end NLC2 GAGTTGTTTGGGAATGCAGCTC	Species per cluster	Cluster number
AF325274	Lactarius	helvus			494	1
AF506411	Lactarius	leonis			494	1
AF325275	Lactarius	lignyotus			494	1
AF325276	Lactarius	lilacinus			494	1
AF325277	Lactarius	necator			494	1
AF325278	Lactarius	pallidus			494	1
AF218554	Lactarius	peckii			494	1
AF325279	Lactarius	piperatus			494	1
AF325280	Lactarius	porninsis			494	1
AY228358	Lactarius	pseudomucidus			494	1
AF325281	Lactarius	pubescens			494	1
AF325282	Lactarius	quieticolor			494	1
AF325284	Lactarius	salmonicolor			494	1
AF325285	Lactarius	scrobiculatus			494	1
AF325286	Lactarius	sp			494	1
AF218555	Lactarius	speciosus			494	1
AF325287	Lactarius	sphagneti			494	1
AF325288	Lactarius	spinosulus			494	1
AF325289	Lactarius	subdulcis			494	1
AF506412	Lactarius	subemboratus			494	1
AF218553	Lactarius	subpurpureus			494	1
AF325290	Lactarius	subsericatus			494	1
AF325291	Lactarius	tabidus			494	1
AF325292	Lactarius	torminosus			494	1
AF325293	Lactarius	uvidus			494	1
AF325294	Lactarius	vellereus			494	1
AF310102	Laxitextum	bicolor			494	1
AF261563	Lentinus	squarrosulus			494	1
AF518627	Lentinus	tigrinus			494	1
LBE487931	Lenzites	betulina			494	1
AF482897	Lepiota	rufipes			494	1
AY586686	Leucogyrophana	romellii			494	1
AF518629	Lindtneria	trachyspora			494	1
AF506441	Megalocystidium	chelidonium			494	1
AF506420	Megalocystidium	leucoxanthu			494	1
AF506422	Megalocystidium	luridum			494	1
AY176456	Melanophyllum	haematospermu			494	1
MGI487930	Meripilus	giganteus			494	1
AF042637	Mycena	clavicularis			494	1
AF261522	Naucoria	bohemica			494	1
AF261517	Naucoria	escharioides			494	1
AY380405	Naucoria	escharoides			494	1
AY261170	Nematoctonus	sp			494	1
U66451	Omphalina	rivulicola			494	1
AF311023	Onnia	tomentosa			494	1
AF287877	Oxyporus	sp			494	1
AF261513	Pachylepyrium	funariophilum			494	1
AB047229	Paecilomyces	pascua			494	1
AY014287	Panellus	serotinus			494	1
AF287878	Panus	rudis			494	1
AF518635	Parmastomyces	transmutans			494	1
AF033397	Penicillium	aculeatum			494	1
AB047230	Penicillium	oblatum			494	1
AF033398	Penicillium	resedanum			494	1
AB047231	Penicillium	sabulosum			494	1
AF033488	Penicillium	soppii			494	1
AF481123	Penicillium	sp			494	1
AF506424	Peniophora	cinerea			494	1
AF506425	Peniophora	incarnata			494	1
PSP406568	Peniophora	sp			494	1
AF329176	Pertusaria	albescens			494	1
AF261505	Phaeocollybia	dissiliens			494	1
AF261506	Phaeocollybia	jennyae			494	1
AF261474	Phaeolepiota	aurea			494	1
AF195599	Phaeonematoloma	myosotis			494	1
AF287883	Phanerochaete	chrysosporium			494	1
PSO406532	Phanerochaete	sordida			494	1
AY059029	Phellinus	cancriformans			494	1
AF311026	Phellinus	chrysoloma			494	1
AF311031	Phellinus	ferrugineofuscus			494	1
AY059020	Phellinus	pachyphloeus			494	1
AF311037	Phellinus	pini			494	1
AY059016	Phellinus	sulphurascens			494	1
AF311044	Phellinus	viticola			494	1
AF141616	Phlebia	albida			494	1
AF141629	Phlebia	serialis			494	1
AF141634	Phlebiopsis	gigantea			494	1
AF195603	Pholiota	aurivella			494	1
AF195606	Pholiota	conissans			494	1

Accession Number	Genus	28S Species	3' end NLB4 GTCATAGAGGGTGAGAATCC	3' end NLC2 GAGTTGTTTGGGAATGCAGCTC	Species per cluster	Cluster number
AF195605	Pholiota	gummosa			494	1
AF195604	Pholiota	jahnii			494	1
AF195594	Pholiota	lignicola			494	1
AF195602	Pholiota	limonella			494	1
AF195607	Pholiota	lundbergii			494	1
AF195609	Pholiota	mixta			494	1
AF261649	Pholiota	oedipus			494	1
AF261642	Pholiota	squarrosa			494	1
AF261641	Pholiota	squarrosoides			494	1
AF195598	Pholiota	subochracea			494	1
PR11925	Phylloporus	rhodoxanthus			494	1
PBE487936	Piptoporus	betulinus			494	1
AY640960	Pleopsidium	chlorophanum			494	1
AY450348	Pleurotus	abieticola			494	1
AY450342	Pleurotus	australis			494	1
PD04158	Pleurotus	dryinus			494	1
AY450347	Pleurotus	eryngii			494	1
POS406580	Pleurotus	ostreatus			494	1
AY450346	Pleurotus	populinus			494	1
AY450349	Pleurotus	pulmonarius			494	1
AF135179	Pleurotus	purpureoolivaceus			494	1
AF135180	Pleurotus	tuberregium			494	1
PAL487937	Polyporus	alveolaris			494	1
PAR487939	Polyporus	arcularius			494	1
PBR487942	Polyporus	brumalis			494	1
PCF488111	Polyporus	cf			494	1
PCI487943	Polyporus	ciliatus			494	1
PGR487946	Polyporus	grammocephalus			494	1
PGU487947	Polyporus	guianensis			494	1
PME487952	Polyporus	meridionalis			494	1
PRH487957	Polyporus	rhizophilus			494	1
PTE488110	Polyporus	tenuiculus			494	1
PTR488112	Polyporus	tricholoma			494	1
PTU488116	Polyporus	tuberaster			494	1
AY059054	Porodaedalea	niemelaei			494	1
AF347093	Porpomyces	mucidus			494	1
AF291266	Protodontia	piceicola			494	1
AF291360	Pseudohydnum	gelatinosum			494	1
AY586701	Pseudomerulius	aureus			494	1
AB104648	Psilocybe	argentipes			494	1
AF141877	Psilocybe	azurescens			494	1
AF261612	Psilocybe	coprophila			494	1
AF261619	Psilocybe	cubensis			494	1
AF261620	Psilocybe	cyanescens			494	1
AB158635	Psilocybe	fasciata			494	1
AF261617	Psilocybe	fimetaria			494	1
AF261618	Psilocybe	liniformans			494	1
AF261613	Psilocybe	merdaria			494	1
AF261614	Psilocybe	moelleri			494	1
AF261611	Psilocybe	pseudobullacea			494	1
AF042567	Psilocybe	stuntzii			494	1
AF261622	Psilocybe	subaeruginosa			494	1
AB104734	Psilocybe	subcubensis			494	1
AB104650	Psilocybe	tampanensis			494	1
AY612821	Pulveroboletus	retipes			494	1
AY059031	Pyrrhoderma	adamanticum			494	1
AY219391	Rhizochaete	americana			494	1
AY219389	Rhizochaete	brunnea			494	1
AY219393	Rhizochaete	filamentosa			494	1
AY219390	Rhizochaete	fouquieriae			494	1
AY219392	Rhizochaete	radicata			494	1
AF335443	Russula	aff			494	1
AF325295	Russula	amoenolens			494	1
AF218548	Russula	brunneola			494	1
AF325297	Russula	caerulea			494	1
AY228344	Russula	cf			494	1
AF325300	Russula	chloroides			494	1
AF287888	Russula	compacta			494	1
AF325301	Russula	cyanoxantha			494	1
AF325303	Russula	delica			494	1
AF325304	Russula	densifolia			494	1
AF325305	Russula	emetica			494	1
AF325306	Russula	exalbicans			494	1
AF325307	Russula	fellea			494	1
AF325308	Russula	firmula			494	1
AF325299	Russula	foetens			494	1
AF218546	Russula	foetentula			494	1
AF325310	Russula	lepida			494	1
AF325311	Russula	mairei			494	1

Accession Number	28S Genus	Species	3' end NLB4 GTCATAGAGGGTGAGAATCC	3' end NLC2 GAGTTGTTTGGGAATGCAGCTC	Species per cluster	Cluster number
AF325312	Russula	nigricans			494	1
AY228349	Russula	occidentalis			494	1
AF506463	Russula	persicina			494	1
AF325315	Russula	puellaris			494	1
AF325317	Russula	raoultii			494	1
AF325318	Russula	sardonia			494	1
AF325319	Russula	solaris			494	1
AF506464	Russula	sphagnophila			494	1
AF325320	Russula	vesca			494	1
AF325321	Russula	veternosa			494	1
AF506465	Russula	violacea			494	1
AF218542	Russula	xerampelina			494	1
AY048164	Saccharomyces	barnettii			494	1
AY130339	Saccharomyces	bayanus			494	1
AF398478	Saccharomyces	cariocanus			494	1
SCRRM01	Saccharomyces	cerevisiae			494	1
AF005707	Saccharomyces	chevalieri			494	1
AY048168	Saccharomyces	dairenensis			494	1
AF005708	Saccharomyces	ellipsoideus			494	1
CHO508565	Saccharomyces	exiguus			494	1
AF398480	Saccharomyces	kudriavzevii			494	1
AY130340	Saccharomyces	kunashirensis			494	1
AY130338	Saccharomyces	pastorianus			494	1
AF406921	Saccharomyces	servazzii			494	1
AB087391	Saccharomyces	sp			494	1
AF398485	Saccharomyces	turicensis			494	1
AY007920	Saccharomyces	unisporus			494	1
SUV279065	Saccharomyces	uvarum			494	1
AF285782	Sagenoma	viride			494	1
AY213628	Scytalidium	hyalinum			494	1
AF506469	Scytinostroma	odoratum			494	1
AF506472	Scytinostromella	nannfeldti			494	1
AF291364	Sebacina	dimitica			494	1
AF440660	Sebacina	endomycorrhiza			494	1
AY505553	Sebacina	vermifera			494	1
SEB534931	Sebacinaceae	sp			494	1
AF518648	Serpula	himantioides			494	1
AY491673	Serpula	incrassata			494	1
SLA440941	Serpula	lacrymans			494	1
AY039334	Stereum	hirsutum			494	1
AF506480	Stereum	reflexulum			494	1
AF506481	Stereum	rugosum			494	1
AF506483	Stereum	sp			494	1
AF506482	Stereum	subtomentosum			494	1
AY341361	Stictis	radiata			494	1
AF195597	Stropharia	aeruginosa			494	1
AF195589	Stropharia	albocrenulata			494	1
AF291368	Stropharia	albonitens			494	1
AY646102	Stropharia	ambigua			494	1
AF261626	Stropharia	umbonescens			494	1
AB047218	Talaromyces	eburneus			494	1
AB047219	Talaromyces	emersonii			494	1
AB047227	Talaromyces	luteus			494	1
AF388747	Thaxterogaster	campbellae			494	1
AF287890	Thelephora	sp			494	1
AF291371	Trametes	gibbosa			494	1
TTR457810	Trametes	trogii			494	1
AY635768	Trechispora	alnicola			494	1
AF347084	Trechispora	araneosa			494	1
AF347081	Trechispora	confinis			494	1
AF347085	Trechispora	incisa			494	1
AF384862	Tremellodendron	pallidum			494	1
AY745701	Tremellodendron	sp			494	1
AF291377	Tremiscus	helvelloides			494	1
TVE406424	Tubulicium	vermiculare			494	1
AY612832	Tylopilus	alboater			494	1
AY612833	Tylopilus	badiceps			494	1
AY612835	Tylopilus	rufonigricans			494	1
AF456813	Tylopilus	sp			494	1
AY612837	Tylopilus	tabacinus			494	1
AY300869	Umbilicaria	crustulosa			494	1
AY645334	Umbilicaria	esculenta			494	1
AY645335	Umbilicaria	mammulata			494	1
AY645337	Umbilicaria	muehlenbergii			494	1
AY645339	Umbilicaria	subglabra			494	1
AY634136	Uncultured	ectomycorrhiza			494	1
AY536734	Uncultured	fungus			494	1
AY452679	Uncultured	mycorrhiza			494	1
AF042563	Unidentified	basidiomycete			494	1

Accession	28S		3' end											NLB4									3' end												NLC2										Species	Cluster
Number	Genus	Species	G	T	C	A	T	A	G	A	G	G	G	T	G	A	G	A	A	T	C	C	G	A	G	T	T	G	T	T	T	G	G	G	A	A	T	G	C	A	G	C	T	C	per cluster	number
AF506484	Vararia	investiens																																											494	1
AY293218	Vararia	sphaericospora																																											494	1
AF261639	Weraroa	virescens																																											494	1
AF514809	Xerocomus	chrysenteron																																											494	1
AF514813	Xerocomus	cisalpinus																																											494	1
AF139712	Xerocomus	depilatus																																											494	1
AF514823	Xerocomus	dryophilus																																											494	1
AF514821	Xerocomus	fennicus																																											494	1
AY612840	Xerocomus	illudens																																											494	1
AF514825	Xerocomus	pruinatus																																											494	1
AF514816	Xerocomus	ripariellus																																											494	1
AY612841	Xerocomus	sp																																											494	1
AY612842	Xerocomus	spadiceus																																											494	1
AF514832	Xerocomus	subtomentosus																																											494	1
AF261462	Xeromphalina	campanelloides																																											494	1
AF261463	Xeromphalina	cornui																																											494	1
AF261464	Xeromphalina	fraxinophila																																											494	1
AY039307	Xylobolus	frustulatus																																											494	1
AY039309	Xylobolus	subpileatus																																											494	1
AF265545	Zelleromyces	sculptisporus																																											494	1
AY075113	Acanthorhynchus	vaccinii		C															G	C							A													T					76	2
AF132319	Annulatascus	hongkongensis		C															G	C							A													T					76	2
AF132320	Annulatascus	velatisporus		C															G	C							A													T					76	2
AF096186	Aporothielavia	leptoderma		C															G	C							A													T					76	2
AF327386	Aschersonia	badia		C															G	C							A													T					76	2
AS17397	Atkinsonella	sp		C															G	C							A													T					76	2
BSU57679	Balansia	strangulans		C															G	C							A													T					76	2
AF339520	Beauveria	caledonica		C															G	C							A													T					76	2
AY346270	Ceratosphaeria	lampadophora		C															G	C							A													T					76	2
AF132328	Cercophora	appalachianensis		C															G	C							A													T					76	2
AF286400	Chaetomium	cupreum		C															G	C							A													T					76	2
CG47825	Chaetomium	globosum		C															G	C							A													T					76	2
CQU312104	Chaetomium	quadrangulatum		C															G	C							A													T					76	2
AJ583471	Cladobotryum	dimorphicum		C															G	C							A													T					76	2
AF160229	Cladobotryum	stereicola		C															G	C							A													T					76	2
AF245294	Claviceps	africana		C															G	C							A													T					76	2
AF009654	Cordycepioideus	bisporus		C															G	C							A													T					76	2
AY283555	Cordyceps	bassiana		C															G	C							A													T					76	2
AB031196	Cordyceps	coccidiicola		C															G	C							A													T					76	2
AB027377	Cordyceps	cochlidiicola		C															G	C							A													T					76	2
AB027365	Cordyceps	jezoensis		C															G	C							A													T					76	2
AY465959	Cordyceps	kyushuensis		C															G	C							A													T					76	2
AY184966	Cordyceps	militaris		C															G	C							A													T					76	2
AB027370	Cordyceps	prolifica		C															G	C							A													T					76	2
AB067738	Cordyceps	sinensis		C															G	C							A													T					76	2
AB027374	Cordyceps	sobolifera		C															G	C							A													T					76	2
AB027375	Cordyceps	sp		C															G	C							A													T					76	2
AB044643	Cordyceps	yakusimensis		C															G	C							A													T					76	2
AY489734	Cosmospora	coccinea		C															G	C							A													T					76	2
AY015625	Cosmospora	episphaeria		C															G	C							A													T					76	2
AY015626	Cosmospora	vilior		C															G	C							A													T					76	2
ETU57083	Echinodothis	tuberiformis		C															G	C							A													T					76	2
AF228356	Fusarium	cavispermum		C															G	C							A													T					76	2
AF130379	Fusarium	dimerum		C															G	C							A													T					76	2
AF228352	Fusarium	epistroma		C															G	C							A													T					76	2
AF228353	Fusarium	melanochlorum		C															G	C							A													T					76	2
FMU88112	Fusarium	merismoides		C															G	C							A													T					76	2
AF228359	Fusarium	sp		C															G	C							A													T					76	2
HT47831	Hirsutella	thompsonii		C															G	C							A													T					76	2
AF213029	Hypomyces	corticiicola		C															G	C							A													T					76	2
AF160238	Hypomyces	mycophilus		C															G	C							A													T					76	2
AF160243	Hypomyces	stephanomatis		C															G	C							A													T					76	2
AY346290	Leptosporella	gregaria		C															G	C							A													T					76	2
MF17404	Melanospora	fallax		C															G	C							A													T					76	2
AF339531	Metarhizium	flavoviride		C															G	C							A													T					76	2
AY554248	Nalanthamala	diospyri		C															G	C							A													T					76	2
AY554259	Nalanthamala	guajavae		C															G	C							A													T					76	2
AY554247	Nalanthamala	sp		C															G	C							A													T					76	2
AY554263	Nalanthamala	vermoesenii		C															G	C							A													T					76	2
NAU88122	Nectria	aurantiaca		C															G	C							A													T					76	2
NCRGND	Nectria	cinnabarina		C															G	C							A													T					76	2
NC17407	Nectria	cosmariospora		C															G	C							A													T					76	2
NEU88100	Nectria	episphaeria		C															G	C							A													T					76	2
NFU88103	Nectria	flammea		C															G	C							A													T					76	2
AY138481	Nectria	mauritiicola		C															G	C							A													T					76	2
NP17410	Nectria	pseudotrichia		C															G	C							A													T					76	2
NPU88099	Nectria	purtonii		C															G	C							A													T					76	2
FVRGN	Nectria	ventricosa		C															G	C							A													T					76	2
NVU57348	Nectria	vilior		C															G	C							A													T					76	2

Accession	28S		3' end	NLB4																			3' end	NLC2																						Species	Cluster
Number	Genus	Species	G	T	C	A	T	A	G	A	G	G	G	T	G	A	G	A	A	T	C	C	G	A	G	T	T	G	T	T	T	G	G	G	A	A	T	G	C	A	G	C	T	C	per cluster	number
NS17423	Nectriopsis	squamulosa		C															G	C							A													T					76	2
AY327047	Neomunkia	sydowii		C															G	C							A													T					76	2
AF539473	Neptunella	longirostris		C															G	C							A													T					76	2
AY489720	Niesslia	exilis		C															G	C							A													T					76	2
AB047211	Nomuraea	rileyi		C															G	C							A													T					76	2
AF172342	Paecilomyces	farinosus		C															G	C							A													T					76	2
AF170081	Paecilomyces	fumosoroseus		C															G	C							A													T					76	2
AF339533	Paecilomyces	javanicus		C															G	C							A													T					76	2
AY554245	Rubrinectria	sp		C															G	C							A													T					76	2
AY015634	Syspastospora	parasitica		C															G	C							A													T					76	2
AF079076	Termitomyces	albuminosus		C															G	C							A													T					76	2
AF339555	Torrubiella	confragosa		C															G	C							A													T					76	2
AY554252	Torrubiella	luteorostrata		C															G	C							A													T					76	2
AF339548	Verticillium	epiphytum		C															G	C							A													T					76	2
AF049176	Verticillium	lecanii		C															G	C							A													T					76	2
AF339563	Verticillium	pseudohemipter		C															G	C							A													T					76	2
AY312603	Verticillium	sp		C															G	C							A													T					76	2
AOV406490	Albatrellus	ovinus				G																																							49	3
AY293166	Albatrellus	subrubescens				G																																							49	3
AY048880	Boidinia	granulata				G																																							49	3
AF506377	Boidinia	macrospora				G																																							49	3
AY684153	Boletinellus	merulioides				G																																							49	3
AF456836	Boletus	aereus				G																																							49	3
AY612800	Boletus	bicolor				G																																							49	3
AF336241	Boletus	radicans				G																																							49	3
AF456824	Boletus	reticulatus				G																																							49	3
AF539721	Cortinarius	amoenus				G																																							49	3
AF311046	Daedaleopsis	confragosa				G																																							49	3
AF310098	Gloeocystidiellum	porosum				G																																							49	3
AF071530	Gomphidius	oregonensis				G																																							49	3
AY612807	Gyrodon	merulioides				G																																							49	3
AY293188	Hyphodontia	barba-jovis				G																																							49	3
AF335441	Lactarius	fallax				G																																							49	3
AF325273	Lactarius	fuliginosus				G																																							49	3
AF218551	Lactarius	fumosus				G																																							49	3
AF325283	Lactarius	ruginosus				G																																							49	3
AF241358	Leaf	litter				G																																							49	3
AF506416	Lentinellus	castoreus				G																																							49	3
AF506417	Lentinellus	cochleatus				G																																							49	3
AF506419	Lentinellus	ursinus				G																																							49	3
AF506423	Metulodontia	nivea				G																																							49	3
AY586691	Mycoacia	aurea				G																																							49	3
AY612815	Paxillus	involutus				G																																							49	3
AY629319	Phaeolus	schweinitzii				G																																							49	3
AF139967	Phanerochaete	chrysorhiza				G																																							49	3
AY645057	Ramaria	rubella				G																																							49	3
AF347098	Ramaria	stricta				G																																							49	3
AF518644	Ramaricium	alboflavescens				G																																							49	3
AY177254	Rhizopogon	pannosus				G																																							49	3
AF071534	Rhizopogon	subcaerulescens				G																																							49	3
AF325296	Russula	atropurpurea				G																																							49	3
AF506427	Russula	aurantiaca				G																																							49	3
AF325298	Russula	cavipes				G																																							49	3
AF325313	Russula	ochroleuca				G																																							49	3
AF325314	Russula	olivacea				G																																							49	3
AF325316	Russula	queletii				G																																							49	3
AF071535	Suillus	cavipes				G																																							49	3
AF347102	Suillus	grevillei				G																																							49	3
AF071536	Suillus	sinuspaulianus				G																																							49	3
AF462350	Suillus	viscidus				G																																							49	3
AF347089	Trechispora	farinacea				G																																							49	3
AF347090	Trechispora	hymenocystis				G																																							49	3
AF347086	Trechispora	kavinioides				G																																							49	3
AF347087	Trechispora	regularis				G																																							49	3
AY647217	Trechispora	sp				G																																							49	3
AY612831	Truncocolumella	citrina				G																																							49	3
AGL312096	Achaetomium	globosum		C			C												G	C							A													T					19	4
AF543792	Aphysiostroma	stercorarium		C			C												G	C							A													T					19	4
AH17400	Atricordyceps	harposporifera		C			C												G	C							A													T					19	4
BO17395	Balansia	obtecta		C			C												G	C							A													T					19	4
BSU68123	Balansia	sclerotica		C			C												G	C							A													T					19	4
AF286396	Chaetomium	brasiliense		C			C												G	C							A													T					19	4
AJ620951	Chaetomium	sp		C			C												G	C							A													T					19	4
AB027373	Cordyceps	heteropoda		C			C												G	C							A													T					19	4
AY544649	Hypocrea	citrina		C			C												G	C							A													T					19	4
AF327387	Hypocrella	discoidea		C			C												G	C							A													T					19	4
HPO459301	Hypomyces	polyporinus		C			C												G	C							A													T					19	4
AF275532	Kernia	geniculotricha		C			C												G	C							A													T					19	4
AF275531	Kernia	hippocrepida		C			C												G	C							A													T					19	4

Accession	28S		3' end NLB4																				3' end NLC2																						Species	Cluster
Number	Genus	Species	G	T	C	A	T	A	G	A	G	G	G	T	G	A	G	A	A	T	C	C	G	A	G	T	T	G	T	T	T	G	G	G	A	A	T	G	C	A	G	C	T	C	per cluster	number
AF275540	Microascus	giganteus		C			C												G	C							A													T					19	4
AF213030	Mycogone	calospora		C			C												G	C							A													T					19	4
AY259543	Polycephalomyces	ramosus		C			C												G	C							A													T					19	4
AF275538	Pseudallescheria	angusta		C			C												G	C							A													T					19	4
AY213623	Pseudallescheria	boydii		C			C												G	C							A													T					19	4
AF373280	Ustilaginoidea	sp		C			C												G	C							A													T					19	4
AF338641	Aspergillus	bombycis					G																																						20	5
AF433048	Aspergillus	caespitosus					G																																						20	5
AM29835	Aspergillus	multicolor					G																																						20	5
AF338647	Aspergillus	nomius					G																																						20	5
AY216673	Aspergillus	puniceus					G																																						20	5
AF433063	Aspergillus	silvaticus					G																																						20	5
AY216676	Aspergillus	ustus					G																																						20	5
AF291298	Basidiodendron	rimosum					G																																						20	5
AY635777	Bourdotia	sp					G																																						20	5
EA29864	Emericella	acristata					G																																						20	5
ED29862	Emericella	dentata					G																																						20	5
ED29838	Emericella	desertorum					G																																						20	5
EF29839	Emericella	fruticulosa					G																																						20	5
EH29871	Emericella	heterothallica					G																																						20	5
EN29859	Emericella	nidulans					G																																						20	5
EP29861	Emericella	parvithecia					G																																						20	5
AY213696	Emericella	quadrilineata					G																																						20	5
ER29860	Emericella	rugulosa					G																																						20	5
ES29845	Emericella	striata					G																																						20	5
EV29858	Emericella	violacea					G																																						20	5
AY327046	Balansia	brunnans		C		G	C												G	C							A													T					12	6
AY489715	Balansia	henningsiana		C		G	C												G	C							A													T					12	6
BNU68119	Balansia	nigricans		C		G	C												G	C							A													T					12	6
AF245293	Balansia	sp		C		G	C												G	C							A													T					12	6
AF178566	Custingophora	olivacea		C		G	C												G	C							A													T					12	6
AY172610	Escovopsis	sp		C		G	C												G	C							A													T					12	6
AY346291	Linocarpon	appendiculatum		C		G	C												G	C							A													T					12	6
AF275539	Microascus	cirrosus		C		G	C												G	C							A													T					12	6
MT47835	Microascus	trigonosporus		C		G	C												G	C							A													T					12	6
MAU68125	Myriogenospora	atramentosa		C		G	C												G	C							A													T					12	6
AB047212	Nomuraea	atypicola		C		G	C												G	C							A													T					12	6
AY494711	Scopulariopsis	flava		C		G	C												G	C							A													T					12	6
AY612812	Boletus	rubropunctus	A																																										11	7
AF261461	Cantharellopsis	prescotii	A																																										11	7
AF347110	Ceriporiopsis	gilvescens	A																																										11	7
AY586645	Chroogomphus	rutilus	A																																										11	7
AF071529	Chroogomphus	vinicolor	A																																										11	7
AY491680	Gyrodontium	sacchari	A																																										11	7
AY586674	Hyphoderma	occidentale	A																																										11	7
AY612810	Leccinum	aurantiacum	A																																										11	7
AF042571	Russula	earlei	A																																										11	7
AY586723	Tylopilus	felleus	A																																										11	7
AF139715	Xerocomus	impolitus	A																																										11	7
AY612816	Phaeogyroporus	sp				G											C																												7	8
AY612822	Phlebopus	beniensis				G											C																												7	8
AY612829	Suillus	americanus				G											C																												7	8
AY612828	Suillus	hirtellus				G											C																												7	8
AF042622	Suillus	luteus				G											C																												7	8
AY684154	Suillus	pictus				G											C																												7	8
AY612826	Suillus	punctipes				G											C																												7	8
AY647211	Boletellus	shichianus					C																																						7	9
AF265534	Cystangium	sp					C																																						7	9
AF452045	Iodosphaeria	sp					C																																						7	9
AF506462	Russula	nauseosa					C																																						7	9
AF325322	Russula	sp					C																																						7	9
AF347080	Trechispora	subsphaerospora					C																																						7	9
AY016369	Trematosphaeria	heterospora					C																																						7	9
AF347105	Basidioradulum	radula	T																																										7	10
AF291338	Hyaloria	pilacre	T																																										7	10
AF291349	Myxarium	grilletii	T																																										7	10
AF291350	Myxarium	mesonucleatum	T																																										7	10
AF291351	Myxarium	nucleatum	T																																										7	10
AF291353	Myxarium	sp	T																																										7	10
AF291352	Myxarium	subhyalinum	T																																										7	10
AB027371	Cordyceps	kanzashiana		C			C												G	C				G			A													T			C		4	11
AB027372	Cordyceps	ramosopulvinata		C			C												G	C				G			A													T			C		4	11
AB044644	Paecilomyces	sp		C			C												G	C				G			A													T			C		4	11
AY259544	Polycephalomyces	formosus		C			C												G	C				G			A													T			C		4	11
AF265531	Arcangeliella	parva							N																																				4	12
MFL406589	Mycena	flavoalba							N																																				4	12
AF218549	Russula	silvicola							N																																				4	12
AF261625	Stropharia	semiglobata							N																																				4	12
AY612804	Boletus	subvelutipes				G	C																																						4	13

Accession Number	28S Genus	Species	3' end NLB4																				3' end NLC2																						Species per cluster	Cluster number
			G	T	C	A	T	A	G	A	G	G	G	T	G	A	G	A	A	T	C	C	G	A	G	T	T	G	T	T	T	G	G	G	A	A	T	G	C	A	G	C	T	C		
AF098398	Gomphidius	glutinosus				G	C																																							4	13
AF356665	Lasallia	pennsylvanica				G	C																																							4	13
AY645059	Paxillus	vernalis				G	C																																							4	13
AY548810	Dendrographa	leucophaea				C														C																										3	14
AY548812	Lecanactis	abietina				C														C																										3	14
AY548815	Schismatomma	decolorans				C														C																										3	14
AF274100	Pertusaria	erythrella			|C|																																							3	15
AF274099	Pertusaria	scaberula			|C|																																							3	15
AY300854	Pertusaria	subventosa			|C|																																							3	15
AY583331	Marchandiomyces	corallinus	A																					C																				G		2	16
AY583332	Marchandiomyces	lignicola	A																					C																				G		2	16
CRO312102	Chaetomium	robustum		C			A												G	C								A														T					2	17
AF396873	Natantispora	lotica		C			A												G	C								A														T					2	17
AY346256	Albertiniella	sp		C															G	C								A		C												T					2	18
AY346283	Helminthosphaeria	clavariar		C															G	C								A		C												T					2	18
AF339518	Aphanocladium	album		C					N										G	C								A														T					2	19
AF339565	Lecanicillium	attenuatum		C					N										G	C								A														T					2	19
AY548814	Roccella	boergesenii		C		C													G	C																										2	20
AY584654	Roccella	fuciformis		C		C													G	C																										2	20
AY016363	Lojkania	enalia					C													C																										2	21
AF284133	Salal	root					C													C																										2	21
AD29795	Aspergillus	deflectus					G												G	C																										2	22
AF433068	Aspergillus	lucknowensis					G												G	C																										2	22
AF398484	Kluyveromyces	sinensis																	|C|																									2	23
AF291358	Protomerulius	africanus																	|C|																									2	23
PGI488126	Pseudotrametes	gibbosa																						C																				G		2	24
AY586703	Pycnoporus	cinnabarinus																						C																				G		2	24
AY219387	Crustoderma	flavescens				T											A																													2	25
AY219388	Crustoderma	longicystidia				T											A																													2	25
AY219386	Crustoderma	cornea	A			C											A																													1	26
AF141633	Phlebia	tristis	A			T											A																													1	27
AY350578	Arthonia	dispersa		C			C				A								G	C																										1	28
AY350580	Combea	mollusca		C			C												G	C																										1	29
AF339535	Rotiferophthora	angustispor		C			C		N										G	C								A														T					1	30
AF440664	Sebacina	sp	|C|																																									1	31
CP47826	Claviceps	paspali		C				T											G	C								A														T					1	32
AY346269	Caudatispora	biapiculata		C		G	C												G	C							C	A														T					1	33
AY227118	Littispora	abonnis		C		G	C		N										G	C								A														T					1	34
VR44092	Varicosporina	ramulosa		C		G	G												G	C								A														T					1	35
AF539469	Sagaaromyces	abonnis		C		G													G	C								A														T					1	36
AY648106	Rhizoplaca	orientalis					C													C										C																	1	37
AF291315	Ductifera	pululahuana					G																											A												1	38
HCI406450	Hyphodontia	cineracea						|A|																																				1	39
AF218544	Russula	adusta						|C|																																				1	40
AY509553	Eichleriella	leveilleana																																						T				1	41
AF139959	Hohenbuehelia	portegna																																				N							1	42
AY174781	Cortinarius	infractus																					|G|																					1	43
AY300870	Umbilicaria	proboscidea																|G|																										1	44
AF005703	Saccharomyces	paradoxus															|C|																											1	45
AF291329	Exidiopsis	sp															|G|																											1	46
AY177250	Rhizopogon	subareolatus							N								A																													1	47
AY509550	Efibulobasidium	rolleyi					|G|																																					1	48
AF279408	Schismatomma	pericleum				C	A												G	C																										1	49
AF274101	Pertusaria	amara				C	C												G																											1	50
AF279400	Mycopepon	smithii				G	C													C																										1	51
AY394689	Uncultured	ascomycete				G	C												G																											1	52
AP29807	Aspergillus	parvulus				G	G																																							1	53
AF279376	Arthrorhaphis	citrinella				G	G												G																											1	54
AF291295	Basidiodendron	cinereum				G																												A												1	55
HAL406453	Hyphodontia	alutacea				N			A																																					1	56
AF071528	Boletus	satanas			|N|																																							1	57
AF291337	Heterochaetella	dubia |K|																																										1	58
AY174810	Cortinarius	scaurus |R|																																										1	59
AY612802	Boletus	edulis	R					G																																						1	60
AF291348	Myxarium	granulum	T																																						T				1	61
AF479200	Abelia	triflora		C		G	G												G	C				G																				C		93	62
AY189029	Actinotus	helianthi		C		G	G												G	C				G																				C		93	62
AY260009	Alangium	chinense		C		G	G												G	C				G																				C		93	62
AY260007	Alangium	kurzii		C		G	G												G	C				G																				C		93	62
AF297544	Alangium	platanifolium		C		G	G												G	C				G																				C		93	62
AF479162	Alangium	sp		C		G	G												G	C				G																				C		93	62
AF479208	Altingia	excelsa		C		G	G												G	C				G																				C		93	62
AY189032	Anginon	rugosum		C		G	G												G	C				G																				C		93	62
AF479222	Aralia	sp		C		G	G												G	C				G																				C		93	62
AY189035	Aralia	spinosa		C		G	G												G	C				G																				C		93	62
AY189037	Arctopus	echinatus		C		G	G												G	C				G																				C		93	62
AY189039	Arthrophyllum	mackeei		C		G	G												G	C				G																				C		93	62
AY189040	Astrantia	major		C		G	G												G	C				G																				C		93	62
AY034040	Athrotaxis	cupressoides		C		G	G												G	C				G																				C		93	62

Accession Number	28S Genus	Species	3' end NLB4 GTCATAGAGGGTGAGAATCC	3' end NLC2 GAGTTGTTTGGGAATGCAGCTC	Species per cluster	Cluster number
AY034042	Athrotaxis	selaginoides	C G G G C	G C	93	62
AY189044	Azorella	macquariensis	C G G G C	G C	93	62
AY189046	Azorella	selago	C G G G C	G C	93	62
AF479246	Barnadesia	caryophylla	C G G G C	G C	93	62
AY292902	Brasenia	schreberi	C G G G C	G C	93	62
AF479112	Brexia	madagascariensis	C G G G C	G C	93	62
AF479191	Campanula	trachelium	C G G G C	G C	93	62
AY260010	Camptotheca	acuminata	C G G G C	G C	93	62
AF389240	Caulophyllum	thalictroides	C G G G C	G C	93	62
AY189054	Centella	erecta	C G G G C	G C	93	62
AF274639	Cercidiphyllum	japonicum	C G G G C	G C	93	62
AY189055	Cheirodendron	trigynum	C G G G C	G C	93	62
AF479190	Codonopsis	pilosula	C G G G C	G C	93	62
AY260011	Cornus	disciflora	C G G G C	G C	93	62
AF297533	Cornus	kousa	C G G G C	G C	93	62
AF297535	Cornus	mas	C G G G C	G C	93	62
AF297539	Cornus	oblonga	C G G G C	G C	93	62
AF297536	Cornus	officinalis	C G G G C	G C	93	62
AF297538	Cornus	racemosa	C G G G C	G C	93	62
AF479213	Corylopsis	pauciflora	C G G G C	G C	93	62
AF274642	Corylopsis	sinensis	C G G G C	G C	93	62
CJU90703	Cryptomeria	japonica	C G G G C	G C	93	62
CLU90699	Cunninghamia	lanceolata	C G G G C	G C	93	62
AY260012	Curtisia	dentata	C G G G C	G C	93	62
AY189057	Cussonia	spicata	C G G G C	G C	93	62
AY189060	Dendropanax	arboreus	C G G G C	G C	93	62
AF205123	Dioscorea	macrostachya	C G G G C	G C	93	62
AF274645	Disanthus	cercidifolius	C G G G C	G C	93	62
AF479189	Donatia	sp	C G G G C	G C	93	62
AY189065	Eleutherococcus	trifoliatus	C G G G C	G C	93	62
AY189068	Eryngium	bourgattii	C G G G C	G C	93	62
AF479113	Euonymus	alatus	C G G G C	G C	93	62
AF479219	Exbucklandia	populnea	C G G G C	G C	93	62
AY189069	Fatsia	japonica	C G G G C	G C	93	62
AY034043	Glyptostrobus	pensilis	C G G G C	G C	93	62
AF479197	Griselinia	lucida	C G G G C	G C	93	62
AY260019	Grubbia	rosmarinifolia	C G G G C	G C	93	62
AF479183	Helianthus	annuus	C G G G C	G C	93	62
AY189074	Heteromorpha	trifoliata	C G G G C	G C	93	62
AF223066	Humulus	lupulus	C G G G C	G C	93	62
AY189076	Hydrocotyle	bowlesioides	C G G G C	G C	93	62
AF479221	Hydrocotyle	sp	C G G G C	G C	93	62
AY189078	Hydrocotyle	verticillata	C G G G C	G C	93	62
AY260021	Hydrostachys	multifida	C G G G C	G C	93	62
AF146016	Ipomoea	lacunosa	C G G G C	G C	93	62
AF148499	Jacquemontia	tamnifolia	C G G G C	G C	93	62
AY189080	Kalopanax	pictus	C G G G C	G C	93	62
AF479206	Lambertia	inermis	C G G G C	G C	93	62
AF274653	Leea	guineensis	C G G G C	G C	93	62
AF479217	Liquidambar	styraciflua	C G G G C	G C	93	62
AF479160	Manilkara	zapota	C G G G C	G C	93	62
AY189085	Melanophylla	alnifolia	C G G G C	G C	93	62
AY034044	Metasequoia	glyptostroboides	C G G G C	G C	93	62
AY189087	Micropleura	renifolia	C G G G C	G C	93	62
AY189088	Motherwellia	haplosciadea	C G G G C	G C	93	62
AY189093	Mulinum	sp	C G G G C	G C	93	62
AF274656	Mytilaria	laosensis	C G G G C	G C	93	62
AF274657	Paeonia	brownii	C G G G C	G C	93	62
AF274658	Paeonia	californica	C G G G C	G C	93	62
AF274659	Paeonia	suffruticosa	C G G G C	G C	93	62
AF274660	Paeonia	tenuifolia	C G G G C	G C	93	62
AF274662	Platanus	occidentalis	C G G G C	G C	93	62
AF222404	Pleurostylia	opposita	C G G G C	G C	93	62
AY189102	Polyscias	guilfoylei	C G G G C	G C	93	62
AY189103	Pseudopanax	arboreus	C G G G C	G C	93	62
AY189107	Sanicula	gregari	C G G G C	G C	93	62
AY189108	Schefflera	arboricola	C G G G C	G C	93	62
SVU90698	Sciadopitys	verticillata	C G G G C	G C	93	62
SSU90701	Sequoia	sempervirens	C G G G C	G C	93	62
AY034045	Sequoiadendron	giganteum	C G G G C	G C	93	62
TCU90700	Taiwania	cryptomerioides	C G G G C	G C	93	62
AY056503	Thuja	plicata	C G G G C	G C	93	62
TGU90696	Torreya	grandis	C G G G C	G C	93	62
AY189113	Torricellia	tiliifolia	C G G G C	G C	93	62
AY189114	Trachymene	coerulea	C G G G C	G C	93	62
AY189115	Trevesia	palmata	C G G G C	G C	93	62
AY189116	Tupidanthus	calyptratus	C G G G C	G C	93	62
AF479199	Valeriana	officinalis	C G G G C	G C	93	62
AF479207	Vitis	aestivalis	C G G G C	G C	93	62

28S

Accession Number	Genus	Species	3' end NLB4																				3' end NLC2																						Species per cluster	Cluster number
			G	T	C	A	T	A	G	A	G	G	G	T	G	A	G	A	A	T	C	C	G	A	G	T	T	G	T	T	T	G	G	G	A	A	T	G	C	A	G	C	T	C		
AF389239	Aextoxicon	punctatum		C			G												G	C				G																			C		43	63
AJ839623	Arabidopsis	thaliana		C			G												G	C				G																			C		43	63
AF389242	Berberidopsis	corallina		C			G												G	C				G																			C		43	63
AY189051	Bowlesia	incana		C			G												G	C				G																			C		43	63
AY189052	Bowlesia	tropaeolifolia		C			G												G	C				G																			C		43	63
D10840	Brassica	napus		C			G												G	C				G																			C		43	63
AF479098	Celtis	yunnanensis		C			G												G	C				G																			C		43	63
AY423091	Chrysothemis	pulchella		C			G												G	C				G																			C		43	63
AY260014	Diplopanax	stachyanthus		C			G												G	C				G																			C		43	63
AF479231	Dipsacus	sativus		C			G												G	C				G																			C		43	63
AY260041	Fendlera	rupicola		C			G												G	C				G																			C		43	63
AF479159	Fouquieria	columnaris		C			G												G	C				G																			C		43	63
AF479158	Galax	urceolata		C			G												G	C				G																			C		43	63
AF479155	Gilia	capitata		C			G												G	C				G																			C		43	63
AF479157	Halesia	diptera		C			G												G	C				G																			C		43	63
AY056515	Impatiens	noli-tangere		C			G												G	C				G																			C		43	63
AF479154	Impatiens	repens		C			G												G	C				G																			C		43	63
AF479165	Justicia	americana		C			G												G	C				G																			C		43	63
AY189082	Mackinlaya	confusa		C			G												G	C				G																			C		43	63
AY189083	Mackinlaya	macrosciadia		C			G												G	C				G																			C		43	63
AY260015	Mastixia	caudatilimba		C			G												G	C				G																			C		43	63
AY260017	Mastixia	eugenioides		C			G												G	C				G																			C		43	63
AY260016	Mastixia	pentandra		C			G												G	C				G																			C		43	63
AF389271	Meliosma	veitchiorum		C			G												G	C				G																			C		43	63
AY260030	Mentzelia	decapetala		C			G												G	C				G																			C		43	63
AY189086	Meryta	sinclairii		C			G												G	C				G																			C		43	63
AY189094	Munroidendron	racemosum		C			G												G	C				G																			C		43	63
AY189098	Panax	quinquefolius		C			G												G	C				G																			C		43	63
AY423082	Peltanthera	floribunda		C			G												G	C				G																			C		43	63
AY260037	Philadelphus	caucasicus		C			G												G	C				G																			C		43	63
AY260036	Philadelphus	hirsutus		C			G												G	C				G																			C		43	63
AF389252	Philadelphus	lewisii		C			G												G	C				G																			C		43	63
AF148281	Phlox	divaricata		C			G												G	C				G																			C		43	63
AF389265	Roupala	macrophylla		C			G												G	C				G																			C		43	63
SARIBRNA	S	alba		C			G												G	C				G																			C		43	63
AF479198	Scabiosa	sp		C			G												G	C				G																			C		43	63
SA25SR	Sinapis	alba		C			G												G	C				G																			C		43	63
AY423092	Sinningia	schiffneri		C			G												G	C				G																			C		43	63
AF479156	Styrax	japonicus		C			G												G	C				G																			C		43	63
AF479153	Tetramerista	sp		C			G												G	C				G																			C		43	63
AY189112	Tetraplasandra	hawaiensis		C			G												G	C				G																			C		43	63
AF479182	Vahlia	capensis		C			G												G	C				G																			C		43	63
AF479130	Viviania	marifolia		C			G												G	C				G																			C		43	63
AY056502	Larix	leptolepis		C		T	G										C		G	C				G																			C		9	64
AY095476	Larix	sp		C		T	G										C		G	C				G																			C		9	64
AY056509	Picea	asperata		C		T	G										C		G	C				G																			C		9	64
AY056510	Picea	breweriana		C		T	G										C		G	C				G																			C		9	64
PCU90681	Pinus	cembra		C		T	G										C		G	C				G																			C		9	64
AY056500	Pinus	mugo		C		T	G										C		G	C				G																			C		9	64
AY056499	Pinus	peuce		C		T	G										C		G	C				G																			C		9	64
AY056501	Pinus	strobus		C		T	G										C		G	C				G																			C		9	64
PMU90682	Pseudotsuga	menziesii		C		T	G										C		G	C				G																			C		9	64
AY189036	Aralidium	pinnatifidum		C		T	G												G	C				G																			C		7	65
AY189041	Astrotricha	pterocarpa		C		T	G												G	C				G																			C		7	65
AY189084	Marianthus	ringens		C		T	G												G	C				G																			C		7	65
AY189097	Osmoxylon	novoguineense		C		T	G												G	C				G																			C		7	65
AY189105	Reynoldsia	sandwicensis		C		T	G												G	C				G																			C		7	65
AY189106	Rhytidosporum	alpinum		C		T	G												G	C				G																			C		7	65
AY189109	Sollya	heterophylla		C		T	G												G	C				G																			C		7	65
AAU90692	Agathis	australis		C		G	G												G	C				G																	A		C		6	66
AAU90690	Araucaria	araucana		C		G	G												G	C				G																	A		C		6	66
AHU90689	Araucaria	heterophylla		C		G	G												G	C				G																	A		C		6	66
FTU90687	Falcatifolium	taxoides		C		G	G												G	C				G																	A		C		6	66
RMU90686	Retrophyllum	minor		C		G	G												G	C				G																	A		C		6	66
WNU90694	Wollemia	nobilis		C		G	G												G	C				G																	A		C		6	66
AGU90683	Abies	grandis		C		T	G										T		G	C				G																			C		4	67
CDU90684	Cedrus	deodara		C		T	G										T		G	C				G																			C		4	67
AY056507	Cedrus	libani		C		T	G										T		G	C				G																			C		4	67
AY056511	Tsuga	canadensis		C		T	G										T		G	C				G																			C		4	67
GBY16380	Ginkgo	biloba		C		C	G												G	C				G																			C		2	68
AY189079	Hymenosporum	flavum		C		C	G												G	C				G																			C		2	68
AF479203	Ilex	opaca		C			A												G	C				G																			C		1	69
AF479223	Myrothamnus	flabellifolius		C		G	G												G	C				G																		N	C		1	71
APU90693	Agathis	palmerstoni	N	C		G	G												G	C				G																	A		C		1	72
